# Targeting the autophagy promoted antitumor effect of T-DM1 on HER2-positive gastric cancer

**DOI:** 10.1038/s41419-020-03349-1

**Published:** 2021-03-17

**Authors:** Jinghui Zhang, Jiajun Fan, Xian Zeng, Mingming Nie, Wei Chen, Yichen Wang, Jingyun Luan, Zeguo Zhu, Xusheng Chang, Dianwen Ju, Li Feng, Kai Yin

**Affiliations:** 1 Department of Gastrointestinal Surgery, Changhai Hospital, Second Military Medical University, Shanghai, 200433 P. R. China; 2 Department of Biological Medicines & Shanghai Engineering Research Center of Immunotherapeutics, Fudan University School of Pharmacy, Shanghai, 201203 P. R. China; 3 Department of Endoscopy Center, Minhang Hospital, Fudan University, 170 Xinsong Road, Shanghai, 201199 P. R. China

**Keywords:** Targeted therapies, Tumour biomarkers

## Abstract

Trastuzumab emtansine (T-DM1), an antibody-drug conjugate consisted of the HER2-targeted monoclonal antibody trastuzumab and the tubulin inhibitor emtansine, has shown potent therapeutic value in HER2-positive breast cancer (BC). However, a clinical trial indicated that T-DM1 exerts a limited effect on HER2-positive gastric cancer (GC), but the underlying mechanism is inconclusive. Our research attempted to reveal the probable mechanism and role of autophagy in T-DM1-treated HER2-positive GC. In this study, our results showed that T-DM1 induced apoptosis and exhibited potent therapeutic efficacy in HER2-positive GC cells. In addition, autophagosomes were observed by transmission electron microscopy. Autophagy was markedly activated and exhibited the three characterized gradations of autophagic flux, consisting of the formation of autophagosomes, the fusion of autophagosomes with lysosomes, and the deterioration of autophagosomes in autolysosomes. More importantly, autophagic inhibition by the suppressors 3-methyladenine (3-MA) and LY294002 significantly potentiated cytotoxicity and apoptosis in HER2-positive GC cells in vitro, while the combined use of LY294002 and T-DM1 elicited potent anti-GC efficacy in vivo. In mechanistic experiments, immunoblot analysis indicated the downregulated levels of Akt, mTOR, and P70S6K and confocal microscopy analysis clearly showed that autophagic inhibition promoted the fusion of T-DM1 molecules with lysosomes in GC cells. In conclusion, our research demonstrated that T-DM1 induced apoptosis as well as cytoprotective autophagy, and autophagic inhibition could potentiate the antitumor effect of T-DM1 on HER2-positive GC. Furthermore, autophagic inhibition might increase the fusion of T-DM1 with lysosomes, which might accelerate the release of the cytotoxic molecule emtansine from the T-DM1 conjugate. These findings highlight a promising therapeutic strategy that combines T-DM1 with an autophagy inhibitor to treat HER-positive GC more efficiently.

## Introduction

Gastric cancer (GC), as the fifth most familiar malignancy worldwide, is the third most common cause of cancer-related mortality^1^. The overexpression of human epidermal growth factor receptor 2 (HER2) is correlated to the pathological mechanism and prognosis of many cancer types, such as breast cancer (BC) and GC^2–5^. It is one of the frequently-used molecular targets for targeted therapy in the clinic, and ~15–20% of GC cases have HER2 overexpression^6,7^. Although HER2-targeted monoclonal antibody drugs have been adopted widely as the targeted therapy for HER2-positive BC, this treatment strategy failed to show significantly therapeutic effect on HER2-positive GC^8^.

Antibody-drug conjugates (ADCs), as promising drugs, conjugate a monoclonal antibody with a cytotoxic agent via a linker. ADCs have shown optimal antitumor efficacy in a series of tumors by simultaneously leveraging the precise targeting properties of the antibody part and the cytotoxicity of the cytotoxic agent^9,10^. For instance, trastuzumab emtansine (T-DM1), an ADC that contains the HER2-targeted monoclonal antibody trastuzumab with the cytotoxic molecule emtansine, can precisely deliver emtansine into HER2-positive tumor cells to achieve antitumor activity and avoid toxicity to healthy tissues^11,12^. Although T-DM1 shows a significant antitumor effect on HER2-positive BC, it achieved only limited therapeutic benefit in HER2-positive GC in a phase II/III clinical study due to primary or acquired therapeutic resistance, which has been confirmed in preclinical models^13,14^. This finding suggested different mechanisms of T-DM1 in HER2-positive GC. Therefore, a deeper exploration of the mechanisms underlying T-DM1 treatment outcomes and potential drug tolerance in HER2-positive GC is urgently needed.

Autophagy, also known as type II programmed cell death, is a conserved intracellular response in eukaryotic cells that balances homeostasis by selectively degrading intracellular components when cells face cellular stress or damage^15^. Autophagy plays context-dependent roles in drug resistance, and appropriate regulation of autophagy can significantly enhance the cell-killing effect of anticancer drugs^16^. In some cases, autophagy may be activated to play a cytotoxic role and thus will be helpful for tumor elimination. However, autophagy exhibits a cytoprotective role in most reported cases, which contributes to the maintenance of cancer cell aggressiveness and drug resistance^17,18^. For instance, it has been reported that activation of cytotoxic autophagy by rapamycin enhances therapeutic efficacy in non-Hodgkin’s lymphoma^19^, while suppression of cytoprotective autophagy enhances the antitumor effects of a series of antitumor agents, such as SIRPα-Fc fusion protein, vismodegib, and arginase^17,20,21^. However, if T-DM1 can induce autophagy and whether it is involved in drug resistance in T-DM1-based therapy for HER2-positive GC remains unclear. Thus, evaluation of the function of autophagy in T-DM1-based GC therapy is useful for revealing the mechanism of low-sensitivity of T-DM1 in HER-positive GC treatment and may provide new insight for developing effective T-DM1-based therapy for HER-positive GC.

In this research, we investigated the antitumor efficacy of T-DM1 in HER2-positive GC in vitro and in vivo. Our results indicated that T-DM1 significantly triggered autophagy in HER2-positive GC. Importantly, T-DM1-induced cytotoxicity and apoptosis were significantly potentiated by pharmacologically inhibiting autophagy in NCI-N87 cells, and the synergistic effect of a combination of T-DM1 and an autophagy inhibitor was further validated in xenograft mice. Moreover, the Akt/mTOR pathway was found to be related to T-DM1-induced autophagy, and inhibition of autophagy promoted the fusion of T-DM1 with lysosomes, the cellular organelles where emtansine is released from the T-DM1 conjugate in GC cells. Together, our findings therefore suggest that autophagy played a cytoprotective role and the combination of T-DM1 and autophagy inhibitors represents an effective strategy to achieve potent therapeutic efficacy for T-DM1-based therapy in HER2-positive GC.

## Results

### Cytotoxicity of T-DM1 in HER2-positive GC cells

**Fig. 1:**
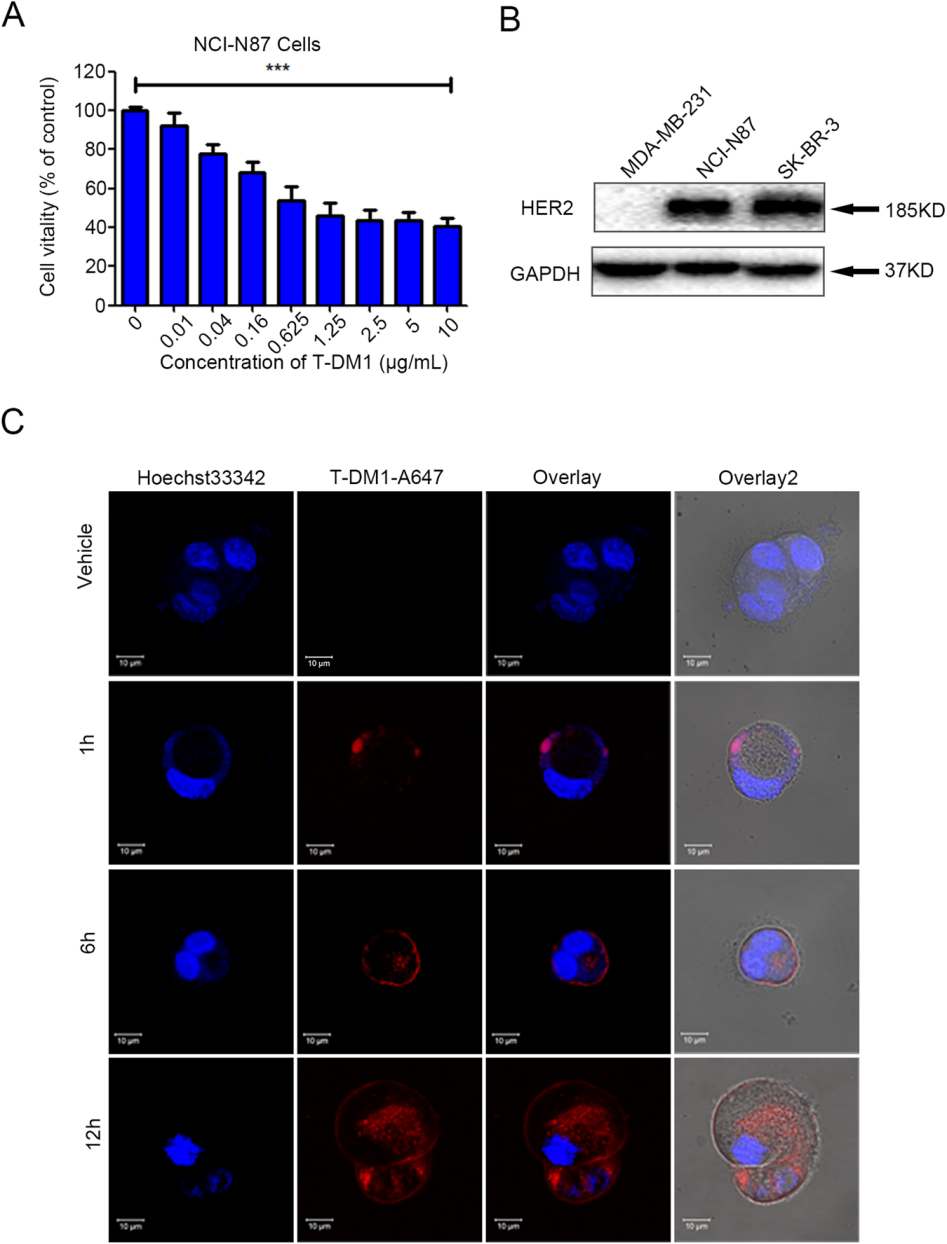
T-DM1 showed antitumor effects in vitro by being internalized into HER2-positive GC cells. **A** NCI-N87 cells were intreated with incremental concentrations of T-DM1 for 72 h and assessed with CCK-8. The results are expressed as the mean ± S.D. (***P < 0.001, *n* = 3). **B** The protein expression level of HER was measured by immunoblot analysis in NCI-N87, MDA-MB-231 (HER2-negative BC), and SK-BR-3 cells (HER2-positive BC). **C** Confocal microscopy experiments showed the whole process of fluorescently labeled T-DM1 internalization in NCI-N87 cells. T-DM1 was labeled with the Alexa Fluor™ 647 Microscale Protein Labeling Kit.

T-DM1 unveiled obvious cytotoxicity in HER2-positive NCI-N87 cells in a dose-dependent manner (Fig. 1A, B). Moreover, the whole process of engulfment of red fluorescence-labeled T-DM1 into these two cell lines was observed (receptor recognition of T-DM1 by HER2 at 1 h, endocytosis of T-DM1 at 6 h, and internalization of T-DM1 at 12 h) under a laser confocal microscope (Fig. 1C).

**Fig. 2:**
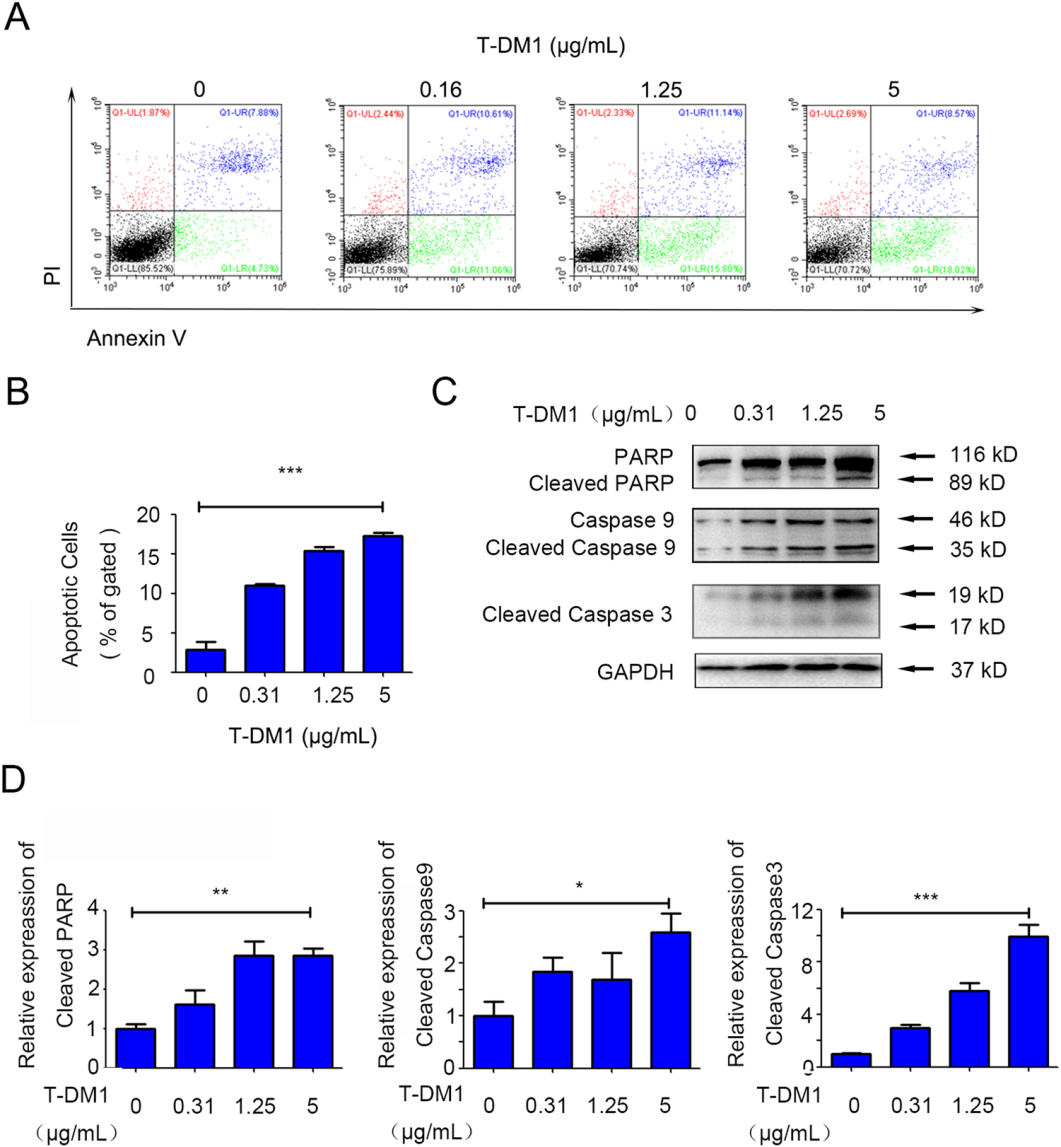
Caspase-dependent apoptosis was induced in both gastric cells by different concentrations of T-DM1. **A** Apoptosis of NCI-N87 cells incubated with T-DM1 for 48 h was evaluated with Annexin V and PI staining and measured by FCM. **B** The percentage of apoptotic cells (Annexin V+) is shown in bar charts. Data are expressed as the mean ± S.D. (***P < 0.001, *n* = 3). **C** HER-positive NCI-N87 cells were incubated with T-DM1 for 48 h. The levels of cleaved PARP and caspase 9/3 were measured by immunoblot analysis. **d** ImageJ software was employed to calculate the densitometric values of NCI-N87 cell samples and normalize the densitometric values to the corresponding control value. The results of the control were set as 1.0, and the values are presented as the mean ± S.D. of 3 independent experiments. Student’s *t*-test. *P < 0.05, **P < 0.01, and ***P < 0.001.

As shown in Fig. 2A and B, the percentage of apoptotic GC cells labeled with Annexin V increased in a dose-dependent manner in NCI-N87 cells treated with T-DM1. Furthermore, caspases 3/9 and PARP dose-dependently activated in NCI-N87 cells, as indicated by immunoblot analysis, which showed that T-DM1 induced conspicuous apoptosis in NCI-N87 cells (Fig. 2C, D).

Thus, these data demonstrated that T-DM1 molecules entered HER2-positive GC cells and induced significant cytotoxicity in vitro.

### T-DM1 induced the formation of autophagosomes and triggered autophagic flux

**Fig. 3:**
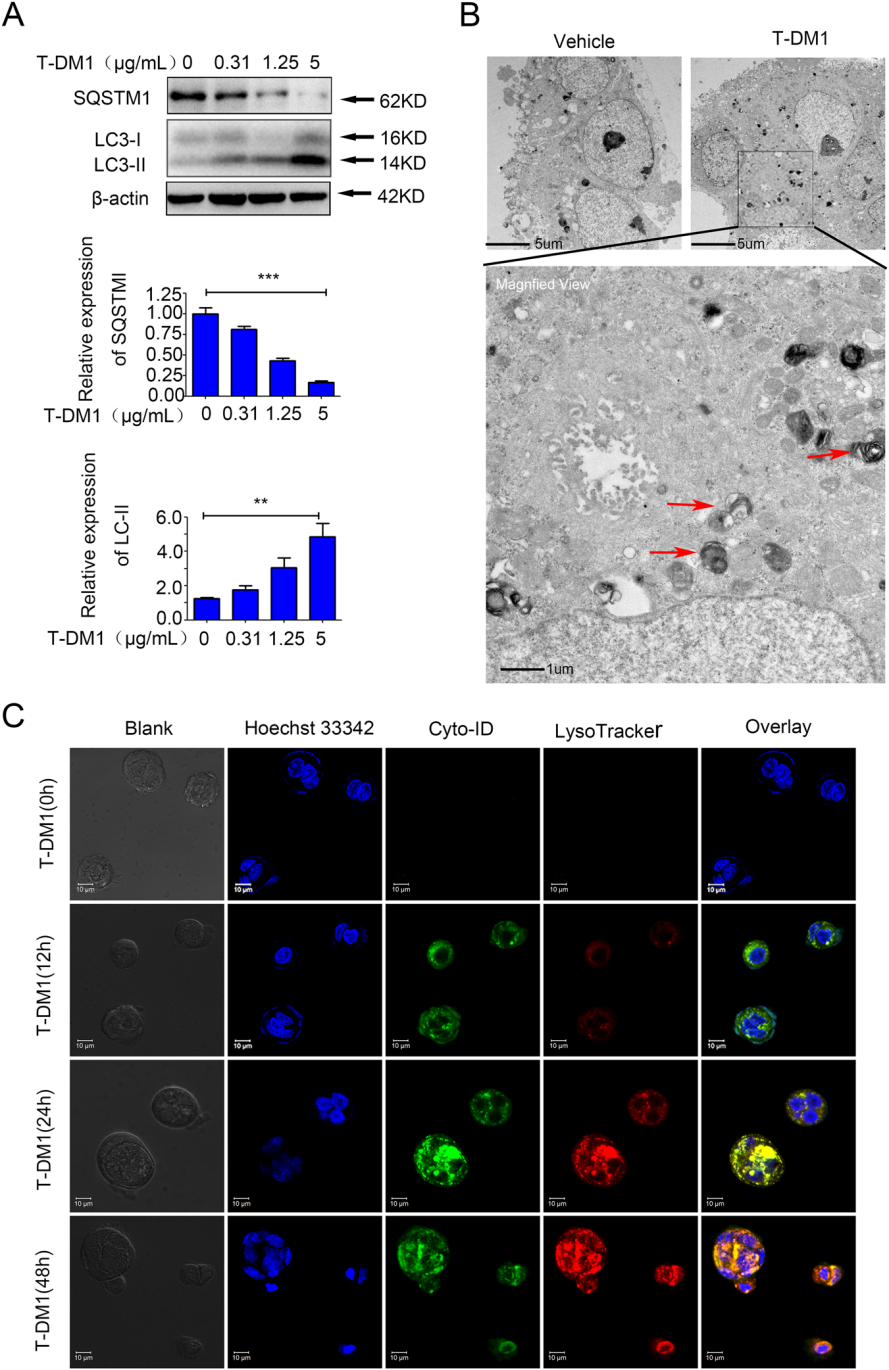
Autophagosomes were induced in HER2-positive GC cells by T-DM1 treatment. **A** NCI-N87 cells were incubated with T-DM1 for 48 h. The levels of SQSTM1 and LC3-II were analyzed by immunoblot analysis. ImageJ software was employed to estimate densitometric values and to normalize the densitometric values to the corresponding control value. The results of control were set as 1.0, and the data are presented as the mean ± S.D. of 3 independent experiments. Student *t*-test was used to compare the data. *P < 0.05, **P < 0.01, and ***P < 0.001. **B** NCI-N87 cells were interfered with T-DM1 for 24 h, and TEM was used to detect ultrastructural autophagosomes shown in the electron photomicrograph. **c** Autophagic flux and lysosomes in NCI-N87 cells treated with T-DM1 for specific times were evaluated by Cyto-ID Green staining and LysoTracker Red staining.

Next, three standard assays were employed to verify the occurrence of autophagy in HER2-positive GC cells after T-DM1 treatment, including immunoblot analysis of microtubule-associated protein 1 light chain 3 (LC3) and sequestosome 1 (SQSTM1, p62), observation of autophagy-specific fluorescence under a confocal microscope, and monitoring of autophagosomes through transmission electron microscope (TEM). Our data showed an upregulated expression level of LC3-II with a downregulated level of SQSTM1 (Fig. 3A), increased punctate autophagosome-like fluorescence, and the formation and the accumulation of double-membraned autophagic vesicles in the cytoplasm (Fig. 3B). TEM analysis showed the formation and aggregation of autophagosomes in the cytoplasm of NCI-N87 cell lines incubated with T-DM1 (Fig. 3B). In addition, analogous to the positive controls treated with rapamycin, NCI-N87 cell lines showed a significant increase in spot-like green fluorescence (autophagosomes) after 24 h of T-DM1 treatment by confocal microscopy (Fig. S2a, b in Supplementary Material).

Moreover, the three critical stages of autophagic flux were also monitored in T-DM1-treated NCI-N87 cells. The results for Cyto-ID and LysoTracker double staining captured the main processes of autophagic flux after T-DM1 treatment: formation of autophagosomes at 12 h (green fluorescence), autophagosomes fusion into lysosomes at 24 h (yellow fluorescence), and degradation of autophagosomes in lysosomes at 48 h (orange fluorescence) (Fig. 3C).

In summary, these results demonstrated that T-DM1 triggered not only autophagosome formation but also autophagic flux in NCI-N87 cells.

### Cytotoxicity and apoptosis were significantly enhanced by blocking T-DM1-induced autophagy in HER2-positive GC cells

**Fig. 4:**
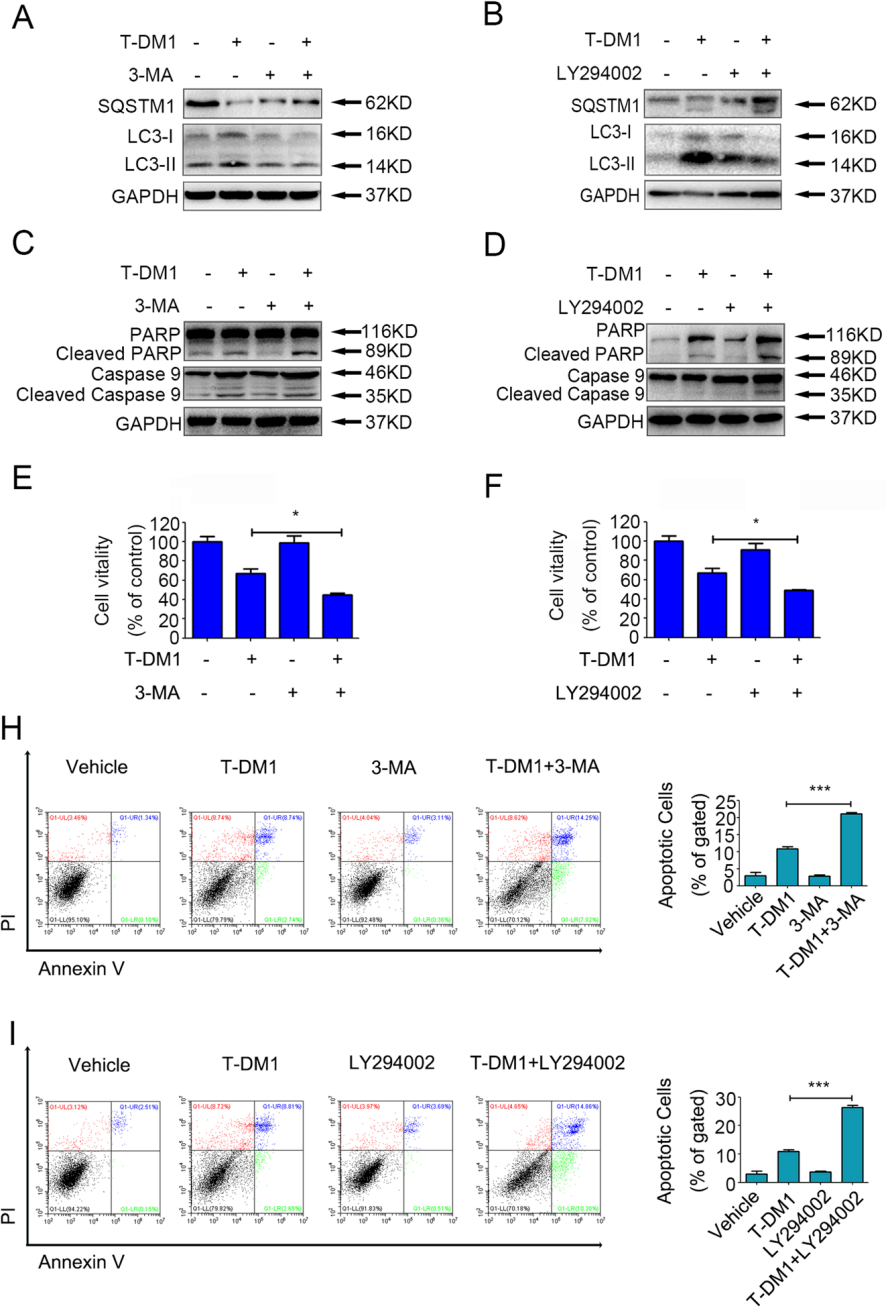
Autophagy was involved in T-DM1-induced cytotoxicity in both HER2-positive GC cells. **A**, **B** NCI-N87 cells were treated with T-DM1 with or without 3-MA and LY294002 for 48 h. The levels of SQSTM1 and LC3-II were measured by immunoblot analysis and the results of statistical analysis were presented in Supplementary Fig. S3a, b. **C**, **D** NCI-N87 cells were treated with T-DM1 with or without 3-MA and LY294002 for 48 h. The levels of cleaved PARP and cleaved caspase 9 were measured by immunoblot analysis and the results of statistical analysis were presented in Supplementary Fig. S3c, d. **E**, **F** The cell viability of NCI-N87 cells incubated with T-DM1 and the autophagic inhibitor 3-MA or LY294002 was detected by a CCK-8 assay. Values are shown as the mean ± S.D. (*P < 0.05, *n* = 3). **H**, **I** The percentages of apoptotic cells following incubation with T-DM1 (0.31 μg/ml) with the autophagic inhibitor 3-MA or LY294002 in NCI-N87 cells were measured by FCM. Apoptotic cells were stained with Annexin V and PI, and the percentage of apoptotic cells (Annexin V + PI− or Annexin V + PI+ cells) is shown in the right bar charts. The values are expressed as the mean ± S.D. (*** versus vehicle, *P* < 0.001, *n* = 3).

To investigate the contribution of autophagy to the T-DM1-induced antitumor effect on NCI-N87 cells in-depth, 3-methyladenine (3-MA) and LY294002 were used to pharmacologically inhibit T-DM1-induced autophagy. As shown in Fig. 4A and Supplementary Fig. 3A and B, compared with T-DM1 treatment alone, cotreatment with 3-MA or LY294002 markedly increased SQSTM1 expression and decreased LC3-II protein expression. In addition, T-DM1-induced cytotoxicity was significantly enhanced by LY294002 or 3-MA in NCI-N87 cell lines (Fig. 4E, F). Similarly, flow cytometry (FCM) studies indicated that T-DM1 combined with LY294002 or 3-MA could significantly induce apoptosis in NCI-N87 cells (Fig. 4H, I), while immunoblot analysis results also showed that T-DM1 combined with LY294002 or 3-MA increased the cleavage of proteins PARP and caspase 9 (Fig. 4C, D; Fig. S3c, d in the Supplementary Material).

Therefore, these results demonstrated that T-DM1-induced autophagy was the cytoprotective mechanism and that cytotoxicity and apoptosis were significantly enhanced by blocking T-DM1-induced autophagy in NCI-N87 cells.

### Inhibiting autophagy enhanced the antitumor efficacy of T-DM1 in vivo

**Fig. 5:**
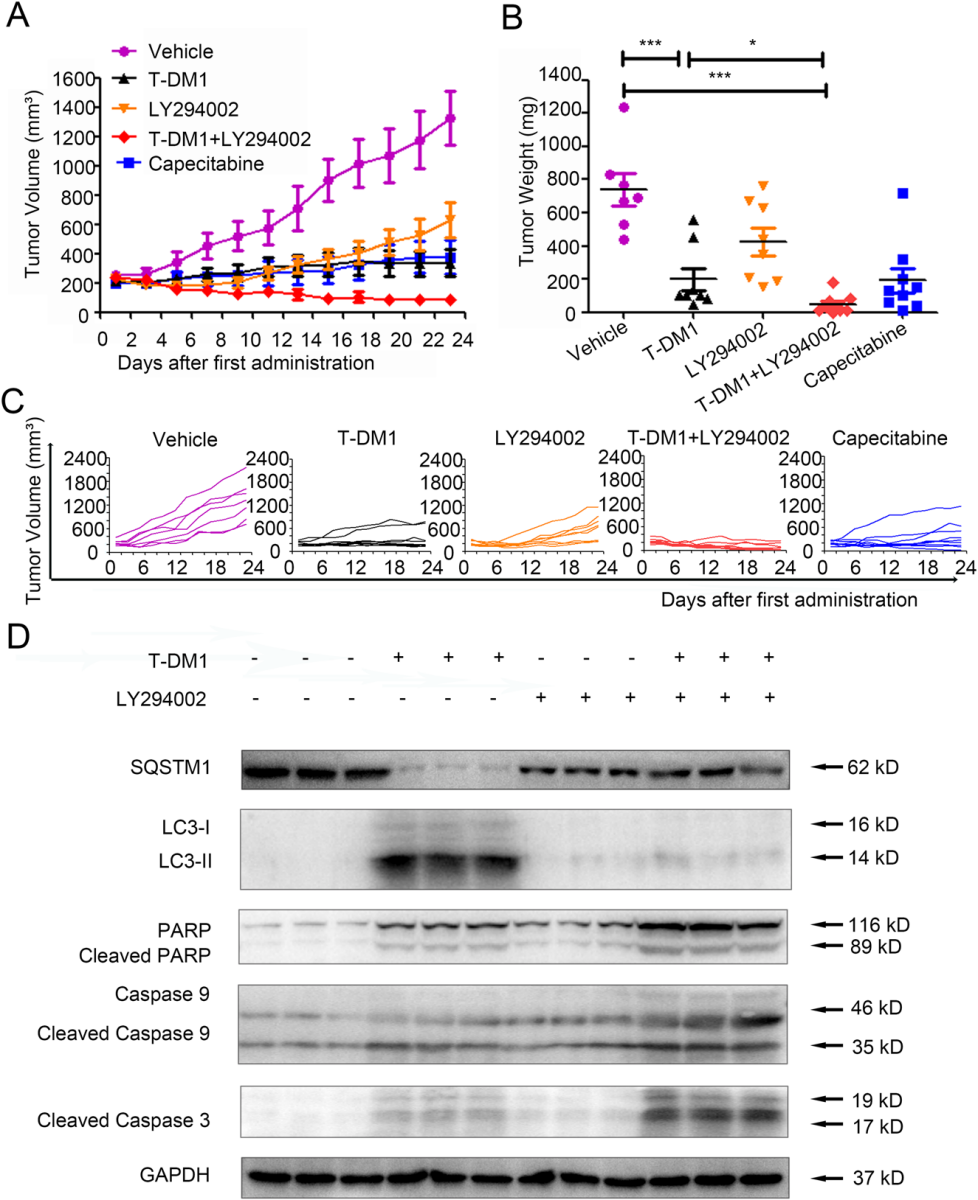
Inhibiting autophagy enhanced the antitumor efficacy of T-DM1 in vivo. **A**–**C** Subcutaneous xenograft models of NCI-N87 cells were established in nude mice. Tumor growth in the different groups was evaluated by caliper measurements and using length × width^2^/2 to calculate tumor volume once every 2 days. Tumor weights were measured after 23 days of intervention, and each point represents an independent data from a mouse. The values of different groups were recorded as the mean ± S.D. The treatment of T-DM1 combined with LY294002 (n = 8) reduced tumor volumes as compared with T-DM1 (*n* = 9) or Vehicle (*n* = 7), *P < 0.05, and ***P < 0.001. T-DM1 treatment alone reduced tumor volumes as compared with Vehicle, ***P < 0.001. **D** The levels of SQSTM1, LC3, cleaved PARP, cleaved caspase 9, and cleaved caspase 3 in xenograft tumor tissue were examined by immunoblot analysis. The results of statistical analysis were presented in Supplementary Fig. S6.

Subcutaneous xenograft models were established with NCI-N87 cells in nude mice. After a 23-day administration of T-DM1 and/or an autophagy inhibitor, the mice were sacrificed, and tumor volume and tumor weight were determined (Fig. 5A–C). Compared with vehicle, mice treated with T-DM1 exhibited an obviously decreased tumor volume, as did mice exposed to capecitabine (positive control). Notably, compared with T-DM1 treatment alone, the result of combining T-DM1 with LY294002 lied in enhanced tumor shrinkage, indicating that inhibiting autophagy promoted the anti-GC efficacy of T-DM1 in vivo (Fig. 5A–C). Specifically, the tumor weight of the T-DM1 group (1 mg/kg, once every 2 days) was 197.50 ± 21.52 mg, while that of the vehicle group was 738.57 ± 36.91 mg on day 23. More importantly, after 23 days of treatment with T-DM1 combined with LY294002, the tumor weight of the mice was 47.78 ± 6.33 mg. Among the 9 experimental mice, one mouse had no tumor, and the tumor weights of 4 mice were <10 mg (Fig. 5B). However, after 23 days of treatment with LY294002 alone or capecitabine alone, the tumor weights of the mice were 425 ± 26.47 mg and 192.22 ± 24.31 mg, respectively, which were larger than those of the combined treatment group (Fig. 5B). As shown in Fig. 5A and C, the tumors of the mice in the T-DM1 combined with LY294002 group grew significantly slower than those of the mice in the T-DM1 group and other groups. By immunoblot analysis, our data showed a decreased expression level of LC3-II with an increased level of SQSTM1, cleaved PARP, and caspase 9/3 in tumor of T-DM1 combined with LY294002 group versus T-DM1-treated group (Fig. 5D).

**Fig. 6:**
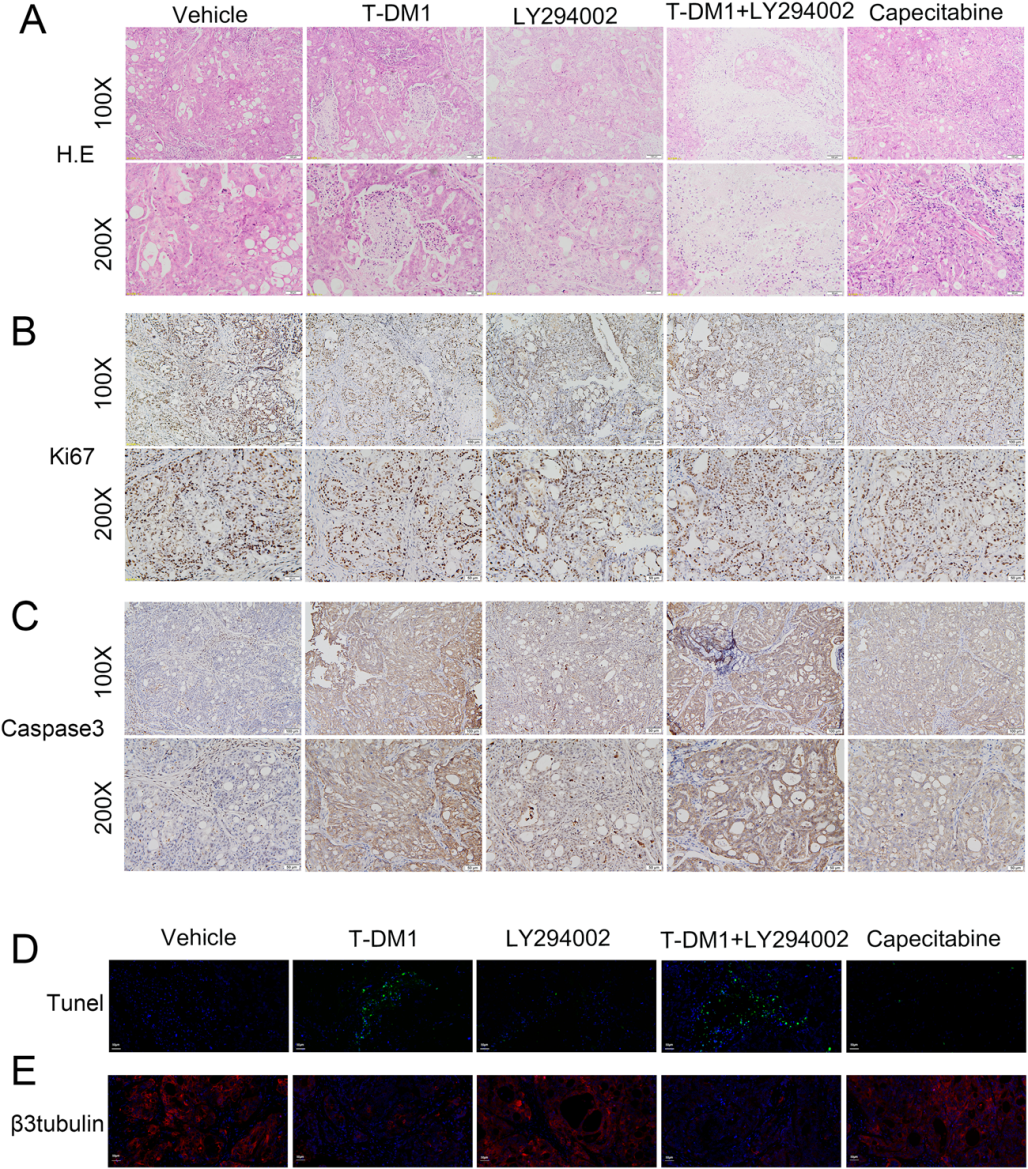
Pathological results for NCI-N87 xenograft tumors. **A** Images of H&E staining of NCI-N87 xenograft tumors showed that treatment with T-DM1 and LY294002. **B**, **C** Images of immunohistochemical staining for Ki67 and caspase 3 in NCI-N87 xenograft tumors presented that treatment with T-DM1 and LY294002. **D**, **E** Images of immunofluorescence TUNEL and β3-tubulin of xenograft tumors from mice treated with T-DM1 with or without LY294002 and the results of statistical analysis were presented in Supplementary Fig. S7.

Histopathological hematoxylin and eosin (H&E) staining showed that treatment with T-DM1 combined with LY294002 increased necrosis in tumor tissue (Fig. 6A). In addition, the tumor tissues in the group of T-DM1 combined with LY294002 could observe an increased expression level of caspase 3 and a decreased expression level of Ki67 (Fig. 6B, C). Similarly, immunofluorescence images showed an intense terminal deoxynucleotidyl transferase-mediated dUTP-biotin nick end labeling (TUNEL) signal in the T-DM1 groups with or without LY294002 (Fig. 6D). Moreover, the expression level of β3-tubulin was significantly decreased in the T-DM1 groups with or without LY294002 (Fig. 6E), which suggested that T-DM1 eliminated GC cells by inhibiting the aggregation of microtubule proteins.

In summary, T-DM1 exhibited a significant antitumor effect, and suppressing autophagy with LY294002 enhanced the antitumor efficacy of T-DM1 in vivo.

### The Akt/mTOR pathway is related to T-DM1-induced autophagy

**Fig. 7:**
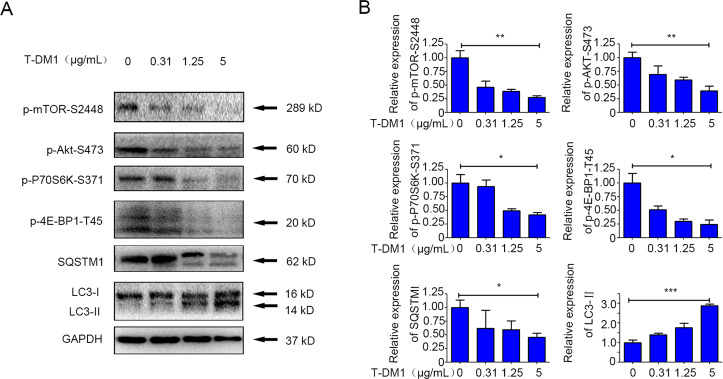
The Akt/mTOR signaling pathway was inactivated by T-DM1 treatment. **A** NCI-N87 cells were cocultured with T-DM1 for 48 h, and immunoblot analysis was applied to examine SQSTM1, LC3, p-Akt, p-mTOR, p-P70S6K, and p-4E-BP1 expression in cell lysates. **B** ImageJ software was used to evaluate the densitometric values of NCI-N87 cells samples and normalize the densitometric values to the corresponding control value. The data of the control were set as 1.0. The results are shown as the mean ± S.D. of 3 independent experiments. Student’s *t*-test. * *P* < 0.05, **P < 0.01, and ***P < 0.001.

To explore the molecular mechanism of the autophagy activated by T-DM1 in NCI-N87 cells, key components of the Akt/mTOR pathway were quantified by immunoblot analysis. The inhibitory effect of T-DM1 on the phosphorylation of S2448 of mTOR was concentration-dependent in the cell lines. Phosphorylation of S473 on Akt, an upstream activator of mTOR, was also shown to be effectively decreased dose-dependently (Fig. 7A, B). Furthermore, 4E-BP1 and p70S6K, both downstream inducers of Akt/mTOR, were also remarkably inhibited (Fig. 7A, B). Besides, we examined Beclin-1, the downstream pathway of mTOR pathway, in gastric cells after T-DM1 treatment. Our results showed that AKT/mTOR pathway upregulated the expression of Beclin-1 protein after T-DM1 treatment, indicating that Beclin-1 might be involved in T-DM1-induced autophagy in GC cells (Fig. S9a, b in the Supplementary Material).

It has been reported that HER2 can indirectly interact with autophagy^22,23^. To further probe the underlying mechanism of T-DM1-induced autophagy, we profiled the transcriptomic changes of T-DM1 treated cells by RNA-seq. The results (Fig. S10a-c in the Supplementary Material) showed that T-DM1 treatment led to a significant increase in expression of autophagy-related genes (ATG14, DEPTOR, and DAPK1). Among these genes, DAPK1 is a positive regulator of both apoptosis and autophagy, and DEPTOR is a negative regulator of mTOR signaling pathway. Projection of these genes on the STRING protein-protein interaction network showed that these genes were centralized in a few STRING subnetworks including “mixed, incl. macroautophagy, and apoptosis (CL:2746)” and a few hub genes (MYC, TP53, BECN1). These clues suggested that BECN1 and mTOR might be involved in T-DM1 induced autophagy in GC cells.

Collectively, our study results showed that the Akt/mTOR pathway was inhibited during the process of T-DM1-induced autophagic initiation and Beclin-1 might be involved in T-DM1-induced autophagy in NCI-N87 cells.

### Autophagy was associated with the fusion of T-DM1 and lysosomes

**Fig. 8:**
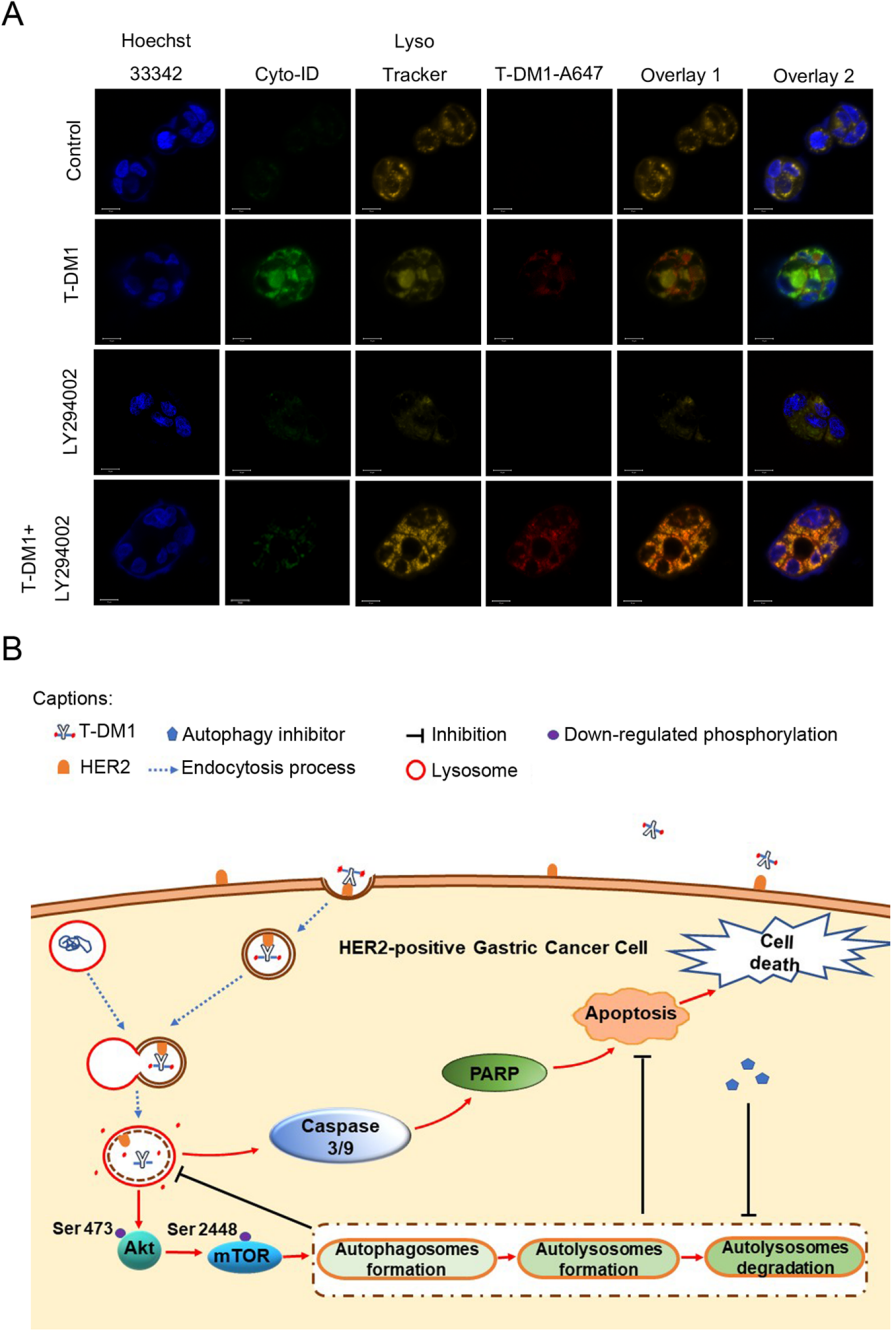
Inhibition of autophagy increases the fusion of T-DM1 and lysosomes. **A** The fusion (orange fluorescence) of fluorescently labeled T-DM1 (red fluorescence) and lysosomes (yellow fluorescence) in NCI-N87 cells visualized by Cyto-ID staining and LysoTracker staining was determined by confocal microscopy after treatment with fluorescently labeled T-DM1 in the presence or absence of LY294002 for 24 h. T-DM1 was labeled with the Alexa Fluor™ 647 Microscale Protein Labeling Kit. **B** A graphical illustration of how T-DM1 and autophagy activation could cause enhanced effects on HER2-positive GC. First, T-DM1 was internalized into cells by targeting the HER2 receptor, and emtansine was released in lysosomes by degradation of the HER2-T-DM1 complex. Moreover, T-DM1 induced cytoprotective autophagy by inactivating the Akt/mTOR signaling pathway and induced apoptosis with activation of caspases 3/9 and PARP. Furthermore, autophagic inhibition could promote the fusion of T-DM1 with lysosomes, significantly enhance apoptosis and augment the therapeutic effect of T-DM1.

To further explore the mechanism of the enhanced T-DM1 antitumor activity resulting from the inhibition of autophagy, we tracked the T-DM1 delivery process in NCI-N87 cells by confocal microscopy. As was well described previously, T-DM1 releases its cytotoxic compound DM1 in lysosomes after it is internalized into cells^11^. Interestingly, confocal microscopy showed that T-DM1 (red fluorescence) and lysosomes (yellow fluorescence) had more pronounced colocalization in NCI-N87 cells after T-DM1 was combined with LY294002 compared with T-DM1 alone, suggesting that inhibiting autophagy increased the release of emtansine in lysosomes via degradation of T-DM1 (Fig. 8A).

In summary, inhibiting autophagy improved the fusion of T-DM1 with lysosomes, which promoted the release of emtansine.

## Discussion

GC, as one of the familiar malignant tumors, leads to 723,000 deaths per year worldwide^24^. HER2-targeted therapy has attracted much attention as a GC therapy due to the considerable occurrence of HER2 overexpression in the GC patient population^6^. Although HER2-targeted methodology has been approved for BC treatment for years, they fail to accomplish desirable therapeutic benefit in HER2-positive GC patients^13,25,26^. T-DM1, the first approved ADC, are released from lysosomes and bind to microtubules to destroy the structure of microtubules and selectively kill HER2-positive malignant tumor cells, but failed to exhibit better antitumor efficacy than taxane (a class of chemotherapeutic drugs for GC) in HER2-positive GC in a phase II/III clinical trial^13,27–29^. These frustrating results highlight the urgent demand for a better understanding of HER2-positive GC.

Our study attempted to address this challenge by investigating the mechanism of autophagy in T-DM1-treated HER2-positive GC. Here, the accumulation of autophagosomes, triggering of autophagic flux as well as upregulation of LC3-II proteins expression confirmed autophagic activation in T-DM1-treated GC cells. In addition, T-DM1 resulted in obvious necrosis, downregulation of Ki67 protein expression, and upregulation of LC3-II protein expressions in tumor tissue in vivo. In previous studies, autophagy has been reported to play an important role in anti-Her2 drugs-based cancer therapies. It has been well documented that trastuzumab could trigger autophagy in both BC cells and BC cell spheroids, and inhibition of autophagy enhanced the antitumor efficacy of trastuzumab, indicating that autophagy plays a cytoprotective role in trastuzumab-based cancer therapy^30,31^. All these data demonstrated that autophagy was activated by T-DM1 in HER2-positive GC, which was first reported here.

However, what effect does autophagy exert in treatment with T-DM1? There is no systematic consensus conclusion on the value of autophagy in cancer therapy^32–34^. Studies have shown that autophagy inhibition destroys the proliferative advantages of hepatocellular carcinoma and glioblastoma^35,36^. Nonetheless, other reports have indicated that the inhibition of resveratrol-induced autophagy decreases the therapeutic benefit of resveratrol in BC^37^. In our research, inhibiting of autophagy effectively increased T-DM1-induced cytotoxicity and apoptosis in vitro and in vivo, indicating that autophagy induced by T-DM1 played a cytoprotective role in HER2-positive GC cells, which is in line with the findings of Jutten B et al., suggesting that the induction of protective autophagy might contribute to the limited effect of T-DM1 on HER2-positive GC^38,39^. In our study, mice were intraperitoneally injected with 1 mg/kg of T-DM1 twice a week when the average tumor volume reached 200 mm^3^ (advanced tumor), while appropriate 3.6 mg/kg of T-DM1 was given as an intravenous infusion over 30 min for a patient with HER2-positive metastatic BC (TNM classification: IV) after prior trastuzumab and taxane treatment once every three weeks and 2.9 mg/kg of T-DM1 was given once every week in high-dose group in clinic, indicating that dose of T-DM1 (used alone) for advanced tumor therapy might be higher than that of its combination use. Moreover, we have demonstrated that intravenously injection of T-DM1 will result in an autophagy-dependent therapy resistance during the process of T-DM1 treatment, which may highlight a potential approach to enhance the antitumor efficacy of T-DM1 in GC therapy.

Previous studies reported some side effects of autophagy inhibitor LY294002, such as rash, tissue growth inhibition, and hyperglycemia. Evidences showed that administration of LY294002 (80 mg/kg for everyday or more) will result in significant adverse events^40,41^. In our animal experiments, 50 mg/kg of LY294002 (twice a week) was used and we did not find severe side effects during the T-DM1 and LY294002 treatment. Therefore, our results might suggest a probable safe dose for LY294002 use in cancer therapy. Besides, our colony assay showed that T-DM1 combining with LY294002 or 3-MA had an effective suppression in colony formation of HER2-positive GC cells, indicating that T-DM1 is equally effective at targeting clonogenic gastric cancer cells and inhibition of autophagy can weaken the cell proliferation ability of HER2-positive GC. This result was consistent with the findings of Diessner et al. (Fig. S8a, b in the Supplementary Material)^29,42^.

The Akt/mTOR signaling pathway plays a vital role in autophagic regulation in eukaryotic cells^43^. When cells are subjected to external damage, autophagy can be induced by downregulating Akt/mTOR pathway activity to sustain cell survival^44^. Our data suggest that inactivation of the Akt/mTOR pathway is related to T-DM1-induced autophagy. More significantly, we found that inhibiting autophagy significantly increased the fusion of T-DM1 molecules with lysosomes, which contributed to the release of emtansine from the T-DM1 conjugate. It is reasonable to deduce that autophagy inhibits the fusion of lysosomes with T-DM1, thereby indirectly inhibiting the release of emtansine via the degradation of T-DM1, which may be one reason for the LY294002-mediated enhancement of the antitumor effect of T-DM1 on HER2-positive GC and supplements the study of resistance to T-DM1 caused by the reduction in lysosomal activity and defects in lysosomal transport^11,14,45^. On that count, the reason T-DM1 failed to exhibit better antitumor efficacy than taxane in phase II/III study of HER2-positive GC might be related to the cytoprotective effect of autophagy. However, these outcomings differed from another study, in which the authors were not convinced that resistance to T-DM1 was related to lysosomal fusion in BC^46^. The differences between the two studies might be due to two reasons. The reason on top of the list is the use of different tumor cell lines. Second, the authors did not detect the change in T‐DM1 fusion with lysosomes by inhibiting autophagy in T‐DM1‐resistant BC cells.

ADCs represent an exciting class of biological drugs that leverage both the precise targeting properties of monoclonal antibodies and the potent cytotoxicity of linked chemical agents^47^. Theoretically, ADCs will achieve better antitumor effects than antibodies or cytotoxic agents used alone. However, there is no guarantee of superior therapeutic advantages in different cancer types^48^. Our study observed the delivery dynamics of T-DM1 from an extracellular medium to intracellular lysosomes, and this process was obviously enhanced by inhibiting of autophagy in HER2-positive GC cells (Fig. 8A). Autophagy delayed the process of T-DM1 entering lysosome, which impaired the antitumor efficacy of the T-DM1. Besides, autophagy could not only contribute to the degradation process of T-DM1, but also probably act as a defender when cells were exposed to emtansine (DM1). Therefore, we supposed that autophagy might play a cytoprotective role in T-DM1-induced cytotoxicity in GC cells. This may contribute to the improved antitumor effect of T-DM1 seen in our study. In clinic, targeted therapies for gastric cancer are facing drug-resistant challenges. T-DM1 was reported to have limited benefits for the GC patients due to complicated mechanisms^13^. Importantly, based on our results, it is reasonable to speculate that autophagy may also interfere with the release of other ADCs in cancer cells, therefore, our study sheds light on the importance of monitoring autophagy in ADC-based cancer therapy. Prospectively, further research on whether autophagy modulation may provide a potential way to reboot the antitumor efficacy of ADCs in insensitive cancer types is warranted.

In conclusion, our study demonstrated that T-DM1 induced autophagy in vitro and in vivo and that the combination of T-DM1 with autophagy inhibitors exhibited a synergistic antitumor effect on HER2-positive GC, which might help to explain the complicated mechanisms of T-DM1 for the GC patients. In addition, our research provided a novel therapeutic approach that combined T-DM1 with autophagic inhibitors for the treatment of HER2-positive GC.

## Materials and methods

### Cell lines and culture

The human GC cell lines NCI-N87 were purchased from the Type Culture Collection of the Chinese Academy of Sciences (Shanghai, China) and were cultured in DMEM medium supplemented with 10% fetal bovine serum, penicillin (100 U/ml), and 100 µg/ml streptomycin (100 µg/ml) at 37 °C with a carbon dioxide content of 5% in constant humidity.

### Reagents and antibodies

The following reagents were purchased: T-DM1 (Roche), LY294002 (Selleck, S1105), 3-MA (Selleck, S2767), and Cyto-ID (Enzo Life Sciences, ENZ-51031-K200). The following major antibodies were obtained for immunoblot analysis: anti-HER (Code: 2165), anti-SQSTM1 (Code: 8025), anti-Beclin-1 (Code: 3495), anti-caspase-9 (Code: 9502), anti-LC3 (Code: 3868), anti-p-Akt (Ser473; Code: 4060), anti-PARP (Code: 9532), anti-GAPDH (Code: 5174), anti-β-actin (Code: 3700), anti-caspase-3 (Code: 9665), anti-phospho-4E-BP1/2/3 (Thr46; Code: 4923), anti-phosphop70S6 kinase (Ser371; Code: 9208), anti-Ki67 (Code: 9449), and anti-p-mTOR (Ser2448; Code: 2971) from Cell Signaling Technology. Horseradish peroxidase (HRP)-conjugated secondary antibodies were obtained from MR Biotech. The major antibodies for immunofluorescence and immunohistochemistry were purchased from Servicebio (Wuhan, China).

### Cell viability assay

Cell viability was determined with Cell Counting Kit-8 (CCK-8). Approximately 10,000 cells/well were inoculated in 96-well plates, while T-DM1 was coincubated with or without the autophagy inhibitor 0.5 mmol/ml of 3-MA or 5 μmol/ml of LY294002 for 72 h. CCK-8 was used immediately according to the relevant process^20^. The absorbance of the surviving cells at a wavelength of 450 nm was determined.

### Transmission electron microscopy

NCI-N87 cells were treated with T-DM1 for 24 h and then prepared immediately according to the relevant process^49^. Cell samples were analyzed with a JEM 1410 transmission electron microscope (JEOL, Inc.). All steps followed the manufacturer’s instructions.

### Immunoblot analysis

Total protein was harvested from cells and subjected to cell lysis buffer for 30 min at 0 °C. The lysates were centrifuged for 10 min and then transferred to Eppendorf tubes. Equal amounts of protein were run by an SDS-PAGE gel and then transferred electrically to a PVDF membrane which was then kept in a solution containing the corresponding primary and secondary antibodies. Finally, the protein content in the immunoreactive bands was visualized using enhanced chemiluminescence reagents, and the intensities of the resulting bands were quantified and compared by ImageJ software (National Institutes of Health, USA).

### Confocal microscopy

NCI-N87 cells were treated with 1.25 µg/mL T-DM1, respectively, for the indicated times, and then stained with Cyto-ID, Hoechst 33342, and/or LysoTracker according to previously described instructions and analyzed with a confocal microscope (Carl Zeiss LSM710, Carl Zeiss, Germany)^50^. Rapamycin (50 nM) was used as the positive control.

### T-DM1 phagocytosis assay

To analyze the phagocytic activity of NCI-N87 cells toward T-DM1, we cocultured them and T-DM1 labeled with the Alexa Fluor™ 647 Microscale Protein Labeling Kit (Code: A30009, Invitrogen) for 0, 1, 6, or 12 h at 37 °C. Then, the cell samples were analyzed with a confocal microscope.

### Apoptosis analysis

The Annexin V-FITC/PI Kit (BD Bioscience, 556547) was used to detect the apoptotic cells of GC. Ten thousand cells were analyzed in each sample after collection, washing, and staining. A FACSCalibur flow cytometer (Becton-Dickinson, Fullerton, CA, USA) was used to analyze each sample.

### Confocal immunofluorescence

HER2-positive GC cells incubated in microscopy chambers and GC tissue samples from a xenograft model were fixed, permeabilized, and blocked with 4% paraformaldehyde, 0.1% Triton X-100, and 10% bovine serum albumin (BSA) for 10 min, 15 min, and 2 h, respectively. These cells or tissue samples were then incubated with anti-HER2, anti-β3-tubulin, and anti-TUNEL antibodies at 4 °C for 12 h and then with PKH26 after washing with phosphate-buffered saline. Finally, the cells or tissue samples were stained with Hoechst 33342, placed on slides or dishes with antifade mounting medium, and then observed with confocal microscopy.

### Xenograft model

BALB/c nude mice were obtained from Beijing Vital River Laboratory Animal Technology Co. Ltd. NCI-N87 cells were collected, and then 1 × 10^7^ cells suspended in DMEM with Matrigel Matrix (BD Bioscience, 356234) were subcutaneously injected to establish a HER-positive GC xenograft model in 8-week-old BALB/c nude mice. The injected mice were randomized and intraperitoneally injected with 1 mg/kg of T-DM1 and 50 mg/kg of LY294002 twice a week when the average tumor volume reached 200 mm^3^. Capecitabine (20 mg/kg) was given twice a week. Every experiment relating to animals complied with procedures approved by the Animal Ethical Committee of the School of Pharmacy of Fudan University.

### Histology and immunohistochemistry

The selected samples of tumor tissues from a xenograft model were fixed, embedded, and stained with 4% paraformaldehyde, paraffin, and H&E staining at a certain temperature to examine tumor necrosis. The remainder slides of tumor tissues were blocked with 3% BSA and incubated with primary antibodies Caspase 3 (1:200) or Ki67 (1:200) for 30 min. Then, the sections were incubated with HRP-conjugated secondary antibodies for 50 min, colored with diaminobenzidine substrate kit (DAKO, K5007), counterstained with Harris hematoxylin for 3 min, and then observed by microscopy. The scores for Caspase 3 and Ki67 immunohistochemistry were calculated according to a previously reported method^51^.

### Colony assay

Approximately 1000 cells/well were inoculated in 6-well plates, while T-DM1 was coincubated with or without the autophagy inhibitor 3-MA or LY294002 for 72 h. Then, NCI-N87 cells could proliferate for 14 days, fixed with 4% paraformaldehyde, and stained with 0.1% crystal violet at a certain temperature and time. Finally, take a picture and count 6-well plates.

### RNA-seq experiments and bioinformatics analysis

Total RNA was extracted from GC cancer cells using TRIzol® Reagent (Invitrogen) according to the manufacturer’s instructions. RNA-seq profiling was performed by Shanghai Majorbio Bio-pharm Technology Co., Ltd (Shanghai, China) using an Illumina Novaseq 6000 sequencer. FastQC package was used to evaluate the quality of raw sequencing files and filtered clean data was subjected to subsequent mapping and quantity analysis pipeline. The kallisto package (v0.46.1) was used to acquire TPM values per transcript from qualified raw files with a kallisto index file built from “Homo_sapiens.GRCh38.96.gtf”. TPM values of genes were summarized from transcripts and genes with <1 TPM across all samples were omitted from subsequent analysis. Genes deferentially expressed between treated samples and control samples were selected according to criteria: a Benjamini–Hochberg adjusted *P*-value <0.05 and the absolute difference is larger than 30% of control. Differentially expressed genes were visualized by heatmap generated using ComplexHeatmap (v1.20.0) R package based on Z-score transformation of log2(TPM + 1) expression matrix. Analysis scripts were coded in R (v3.5.0). All analyses were run on an Ubuntu 18.04 server station with 16-cores CPU and 64GB RAM storage. The network representation of selected genes was generated using STRING database by allowing <5 connected nodes.

### Statistical analysis

All data were analyzed with GraphPad Prism 5 (GraphPad Software Inc., San Diego, CA). The mean ± S.D. is used to represent results. Student’s *t*-test were used for all statistical analyses. When *P* < 0.05, differences were deemed statistically significant.

### Supplementary information

Supplementary Material Legends

Supplementary Figure S1

Supplementary Figure S2

Supplementary Figure S3

Supplementary Figure S4

Supplementary Figure S5

Supplementary Figure S6

Supplementary Figure S7

Supplementary Figure S8

Supplementary Figure S9

Supplementary Figure S10

Related Manuscript File
