# Correction: Targeting the autophagy promoted antitumor effect of T-DM1 on HER2-positive gastric cancer

**DOI:** 10.1038/s41419-023-06061-y

**Published:** 2023-09-26

**Authors:** Jinghui Zhang, Jiajun Fan, Xian Zeng, Mingming Nie, Wei Chen, Yichen Wang, Jingyun Luan, Zeguo Zhu, Xusheng Chang, Dianwen Ju, Li Feng, Kai Yin

**Affiliations:** 1 Department of Gastrointestinal Surgery, Changhai Hospital, Second Military Medical University, Shanghai, 200433 PR China; 2 Department of Biological Medicines & Shanghai Engineering Research Center of Immunotherapeutics, Fudan University School of Pharmacy, Shanghai, 201203 PR China; 3 Department of Endoscopy Center, Minhang Hospital, Fudan University, 170 Xinsong Road, Shanghai, 201199 PR China

**Keywords:** Targeted therapies, Tumour biomarkers

Correction to: *Cell Death & Disease* 10.1038/s41419-020-03349-1, published online 17 March 2021

In this article the caption of Fig. 1 is given erroneously. It should read:

**Fig. 1 T-DM1 showed antitumor effects in vitro by being internalized into HER2-positive GC cells.** **A** NCI-N87 cells were intreated with incremental concentrations of T-DM1 for 72 h and assessed with CCK-8. The results are expressed as the mean ± S.D. (***P < 0.001, *n* = 3). **B** The protein expression level of HER was measured by immunoblot analysis in NCI-N87, MDA-MB-231 (HER2-negative BC), and SK-BR-3 cells (HER2-positive BC). **C** Confocal microscopy experiments showed the whole process of fluorescently labeled T-DM1 internalization in NCI-N87 cells. T-DM1 was labeled with the Alexa Fluor™ 647 Microscale Protein Labeling Kit.

The original article has been corrected.

