# Supplementary material for: Targeting the autophagy promoted antitumor effect of T-DM1 on HER2-positive gastric cancer
Source: Cell Death Dis. 2021 Mar 17;12(4):288. doi: 10.1038/s41419-020-03349-1 (PMC7969610; doi:10.1038/s41419-020-03349-1)
Supplement: Supplementary file 1 — Supplementary Material Legends [file 41419_2020_3349_MOESM1_ESM.docx]

Supplementary Material

**Supplementary Figure S1.** The protein expression level of HER was measured by immunofluorescence assays in NCI-N87, MDA-MB-231 (HER2-positive BC) and SK-BR-3 cells (HER2- negative BC).

**Supplementary Figure S2**. **a** Autophagy in NCI-N87 cells after treatment with T-DM1 for 24 h was evaluated by Cyto-ID Green staining and Hoechst 33342 Blue staining. The standard control was rapamycin. **b** ImageJ software was employed to estimate brightness values of Cyto-ID Green staining and to normalize the densitometric values to the corresponding control value. The values of control were set as 1.0, and the data were presented as the mean ± S.D. of 3 independent experiments. Student’s t-test was used to compare the data. ***P* < 0.01 and ****P* < 0.001. **c** ImageJ software was used to evaluate the brightness values of autophagic flux and to normalize the values to the corresponding control value. The values of the control were set as 1.0. Data were shown as the mean ± S.D. of 3 independent experiments and were compared by Student’s t-test. ***P* < 0.01 and ****P* < 0.001.

**Supplementary Figure S3.** **a, b** Densitometric values of SQSTM1 and LC-Ⅱ in NCI-N87 cells treated with T-DM1 with or without LY294002/3-MA. Data were shown as the mean ± S.D. of 3 independent experiments and were compared by Student’s t-test. **P* < 0.05, ***P* < 0.01, and ****P* < 0.001. **c, d** ImageJ software was used to evaluate the densitometric values of PARP and Cleaved Caspase9 in NCI-N87 cells treated with T-DM1 with or without LY294002/3-MA. Data were shown as the mean ± S.D. of 3 independent experiments and were compared by Student’s t-test. **P* < 0.05 and ***P* < 0.01.

**Supplementary Figure S4**. Photos of NCI-N87 xenograft tumors treated with T-DM1 with or without LY294002.

**Supplementary Figure S5**. Weights of NCI-N87 xenograft mice treated with T-DM1 with or without LY294002.

**Supplementary Figure S6**. ImageJ software was used to evaluate the densitometric values of SQSTMI, LC-Ⅱ, cleaved PARP, cleaved Caspase9 and cleaved Caspase3, and to normalize the densitometric values to the corresponding control value. The values of the control were set as 1.0. Data were shown as the mean ± S.D. of 3 independent experiments and were compared by Student’s t-test. **P* < 0.05 and ***P* < 0.01.

**Supplementary Figure S7. a, b** ImageJ software was used to evaluate the brightness values of Tunel and β3tubulin, and to normalize the densitometric values to the corresponding control value. The values of the control were set as 1.0. Data were compared by Student’s t-test. n=3, **P* < 0.05, and ***P* < 0.01.

**Supplementary Figure S8** **T-DM1 combining with LY294002 or 3-MA influences clonogenic gastric cancer cells. a** NCI-N87 cells were cultured for 14 days after cocultured with 0.08 µg/ml T-DM1 with or without 0.5mmol/ml 3-MA for 72 h. The values of the control were set as 1.0, and data were compared by Student’s t-test. n=3, ****P* < 0.001. **b** NCI-N87 cells were cultured for 14 days after cocultured with 0.08 µg/ml T-DM1 with or without 5μmol/ml LY294002 for 72 h. The values of the control were set as 1.0. Data were compared by Student’s t-test. n=3, ****P* < 0.001.

**Supplementary Figure S9. a** NCI-N87 cells were cocultured with T-DM1 for 48 h, and immunoblot analysis was applied to examine Beclin-1 expression in cell lysates. **b** ImageJ software was used to evaluate the brightness values of Beclin-1, and to normalize the densitometric values to the corresponding control value. The values of the control were set as 1.0, and data were compared by Student’s t-test. n=3, ***P* < 0.01.

**Supplementary Figure S10.** Six GC cell samples were treated with T-DM1 at 1.25 µg/ml for 48 hours. Total RNA was extracted and sequenced. **a** Multidimensional scaling (MDS) plot of samples based on gene expression values (TPM) of all detected genes to show the distribution of sequenced samples. **b** Heatmap was generated to visualize the expression profile of differentially expressed genes between T-DM1 treated samples and control samples. **c** Differentially expressed genes and their interaction neighbors in Protein-Protein interaction networks were analyzed using STRING database. Control samples: C1, C2, and C3; T-DM1 treated samples: T1, T2, and T3.
