# Supplementary figures and images for: Targeting the autophagy promoted antitumor effect of T-DM1 on HER2-positive gastric cancer
Source: Cell Death Dis. 2021 Mar 17;12(4):288. doi: 10.1038/s41419-020-03349-1 (PMC7969610; doi:10.1038/s41419-020-03349-1)

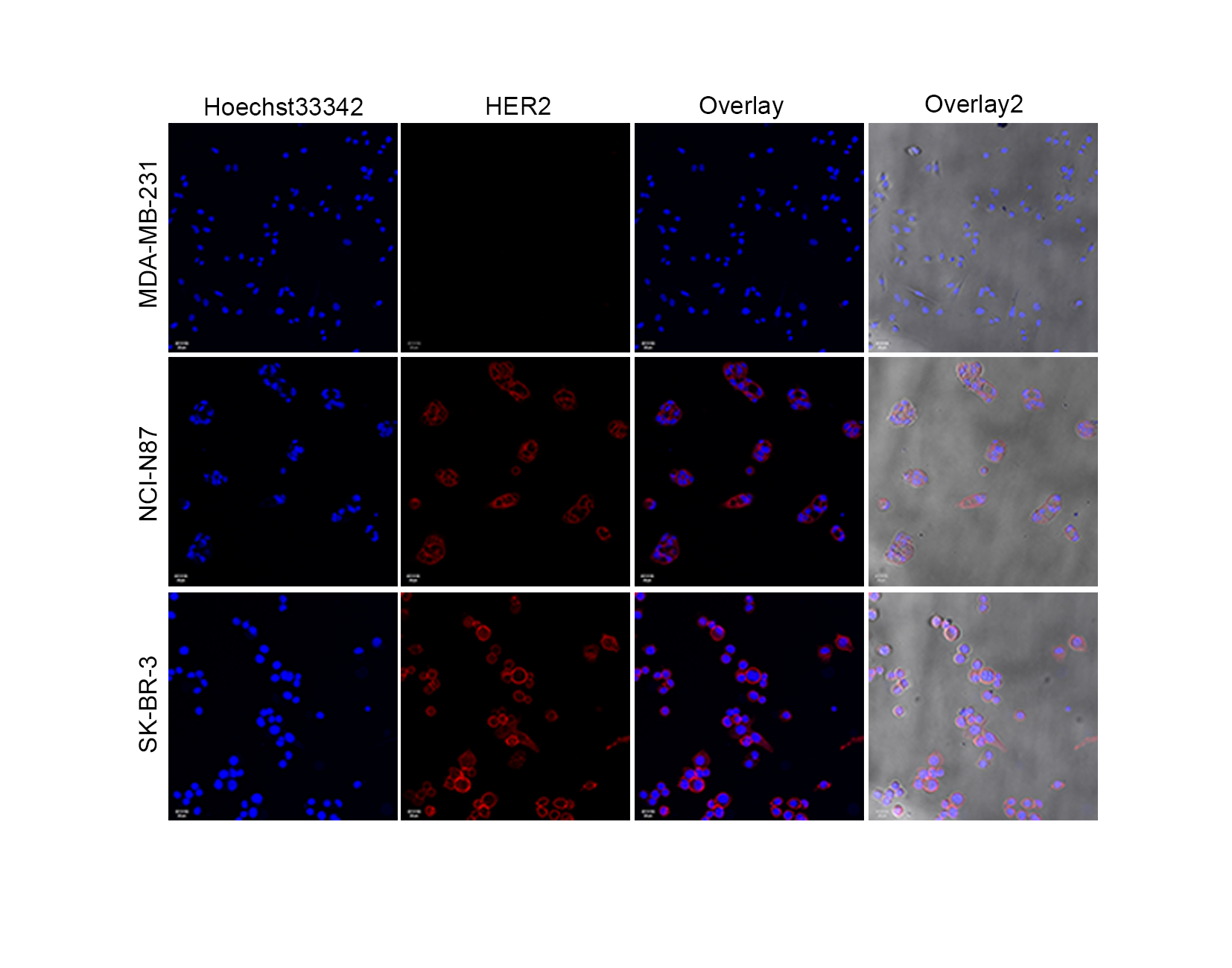

Supplement: Supplementary file 2 — Supplementary Figure S1 [file 41419_2020_3349_MOESM2_ESM.tif]

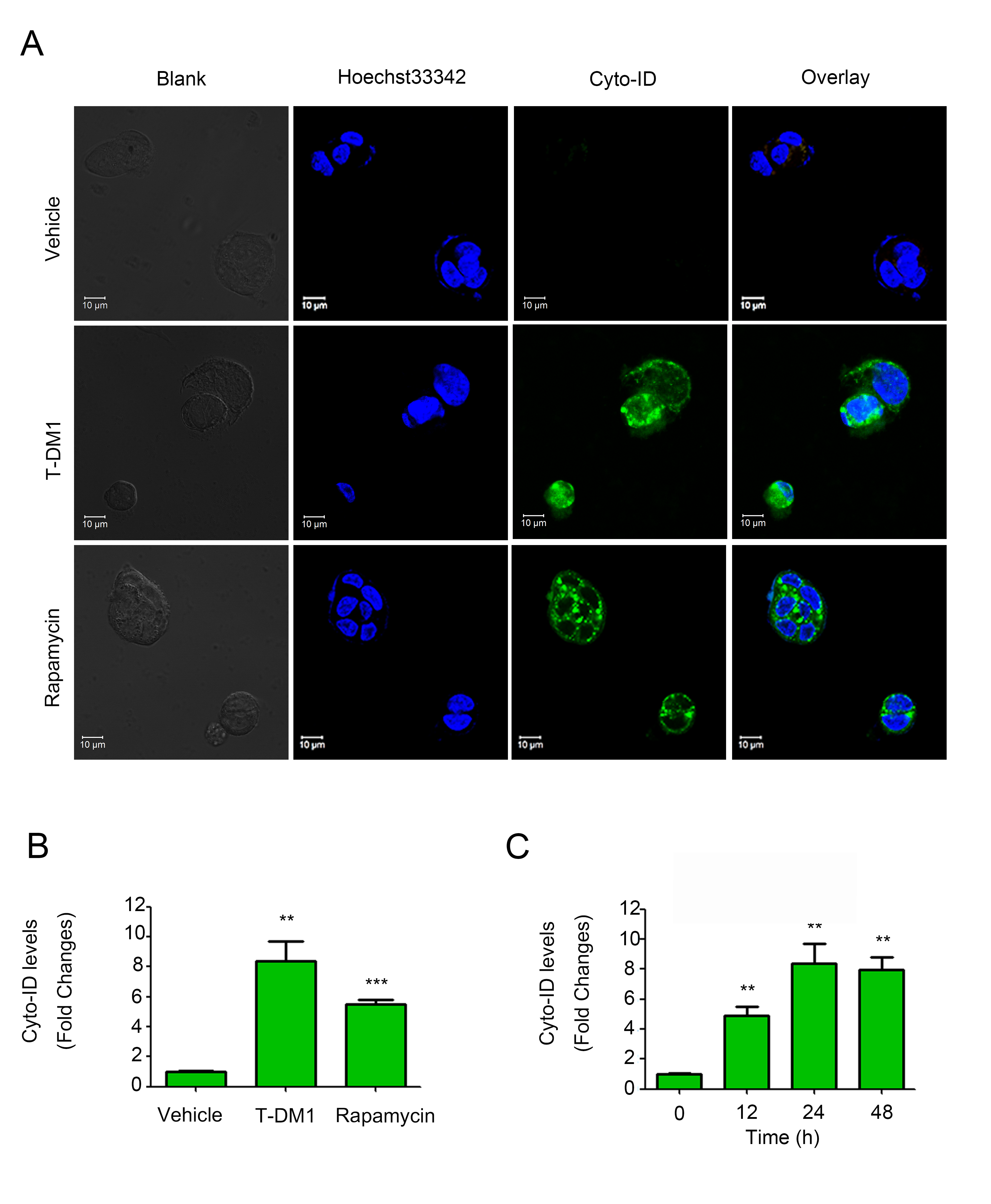

Supplement: Supplementary file 3 — Supplementary Figure S2 [file 41419_2020_3349_MOESM3_ESM.tif]

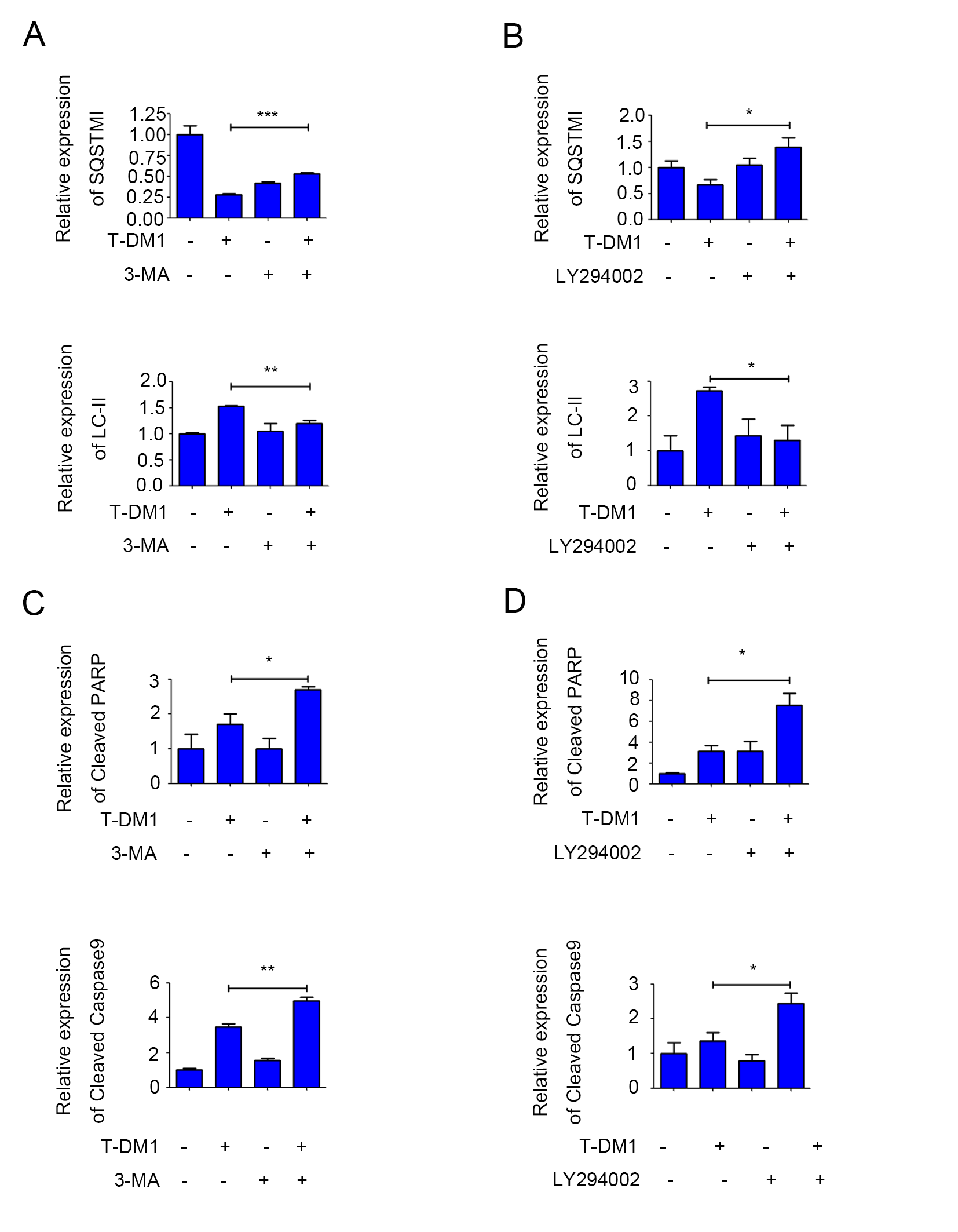

Supplement: Supplementary file 4 — Supplementary Figure S3 [file 41419_2020_3349_MOESM4_ESM.tif]

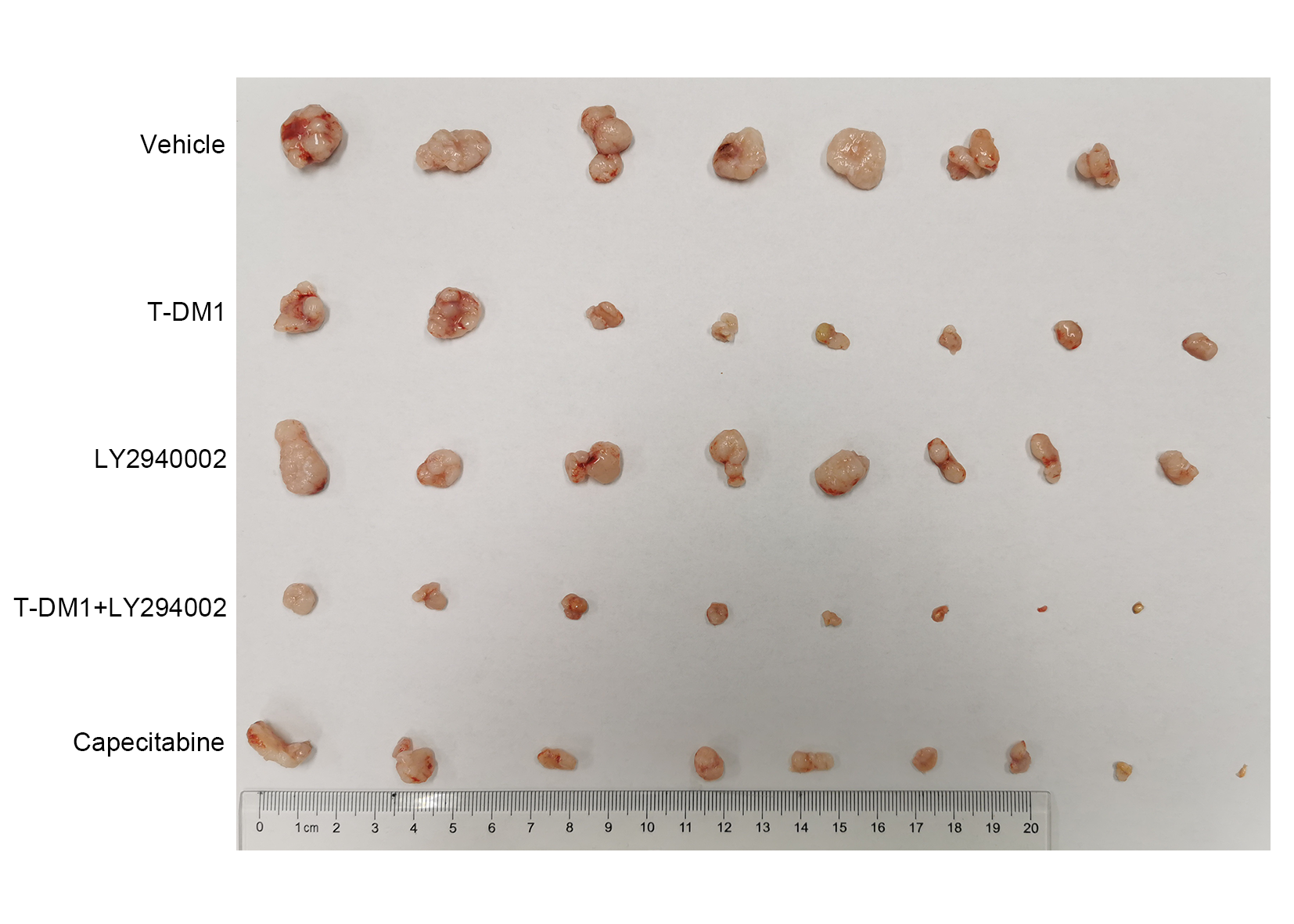

Supplement: Supplementary file 5 — Supplementary Figure S4 [file 41419_2020_3349_MOESM5_ESM.tif]

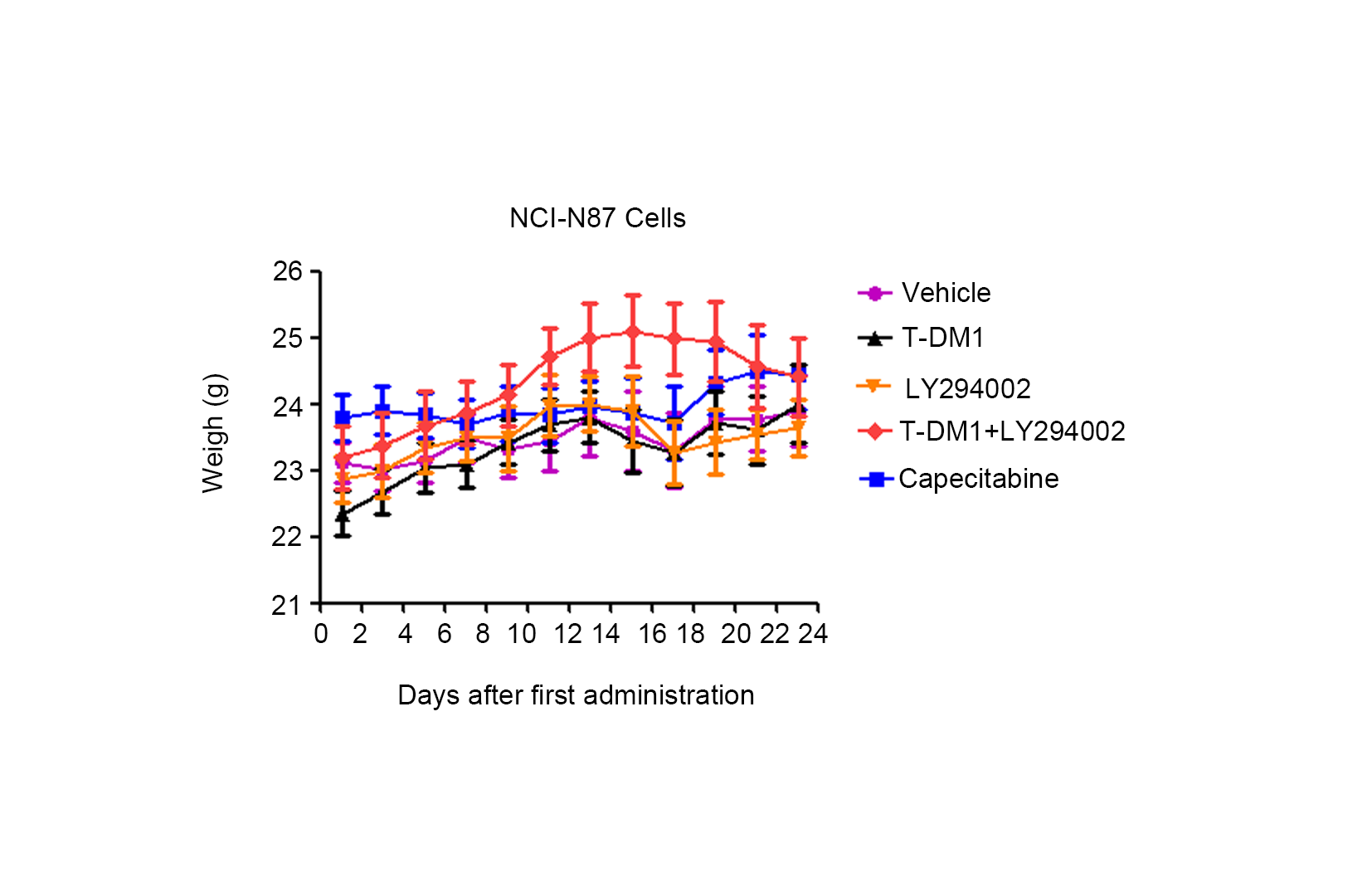

Supplement: Supplementary file 6 — Supplementary Figure S5 [file 41419_2020_3349_MOESM6_ESM.tif]

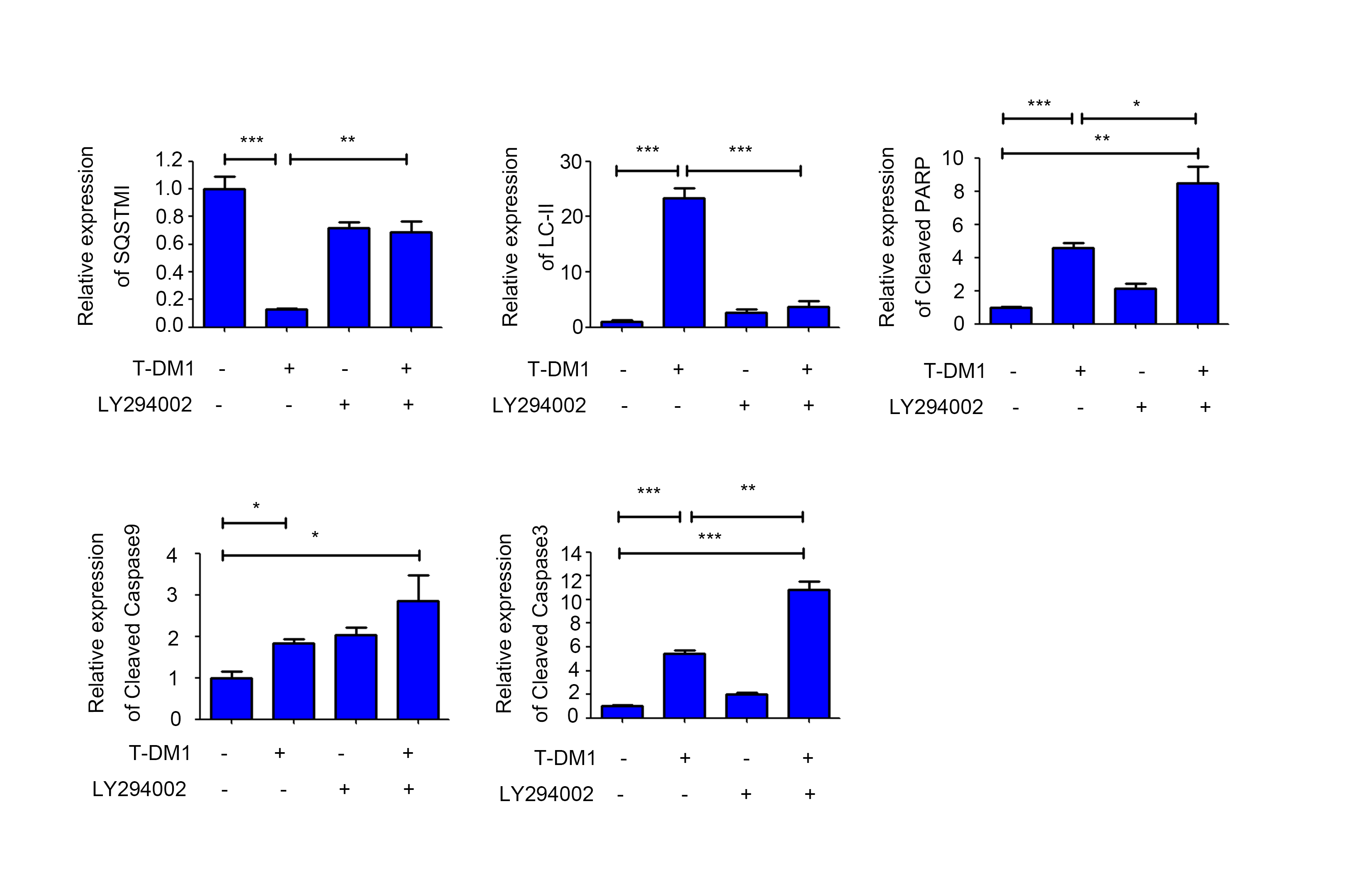

Supplement: Supplementary file 7 — Supplementary Figure S6 [file 41419_2020_3349_MOESM7_ESM.tif]

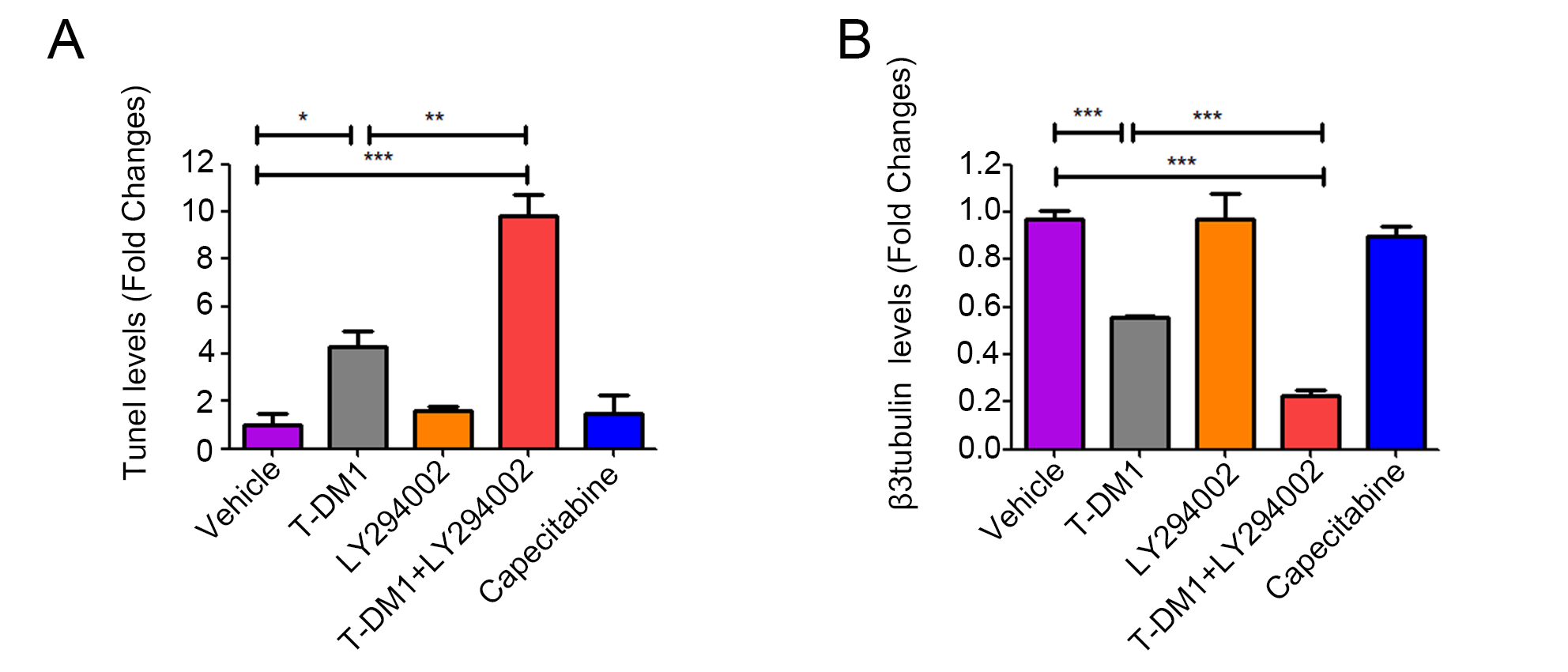

Supplement: Supplementary file 8 — Supplementary Figure S7 [file 41419_2020_3349_MOESM8_ESM.tif]

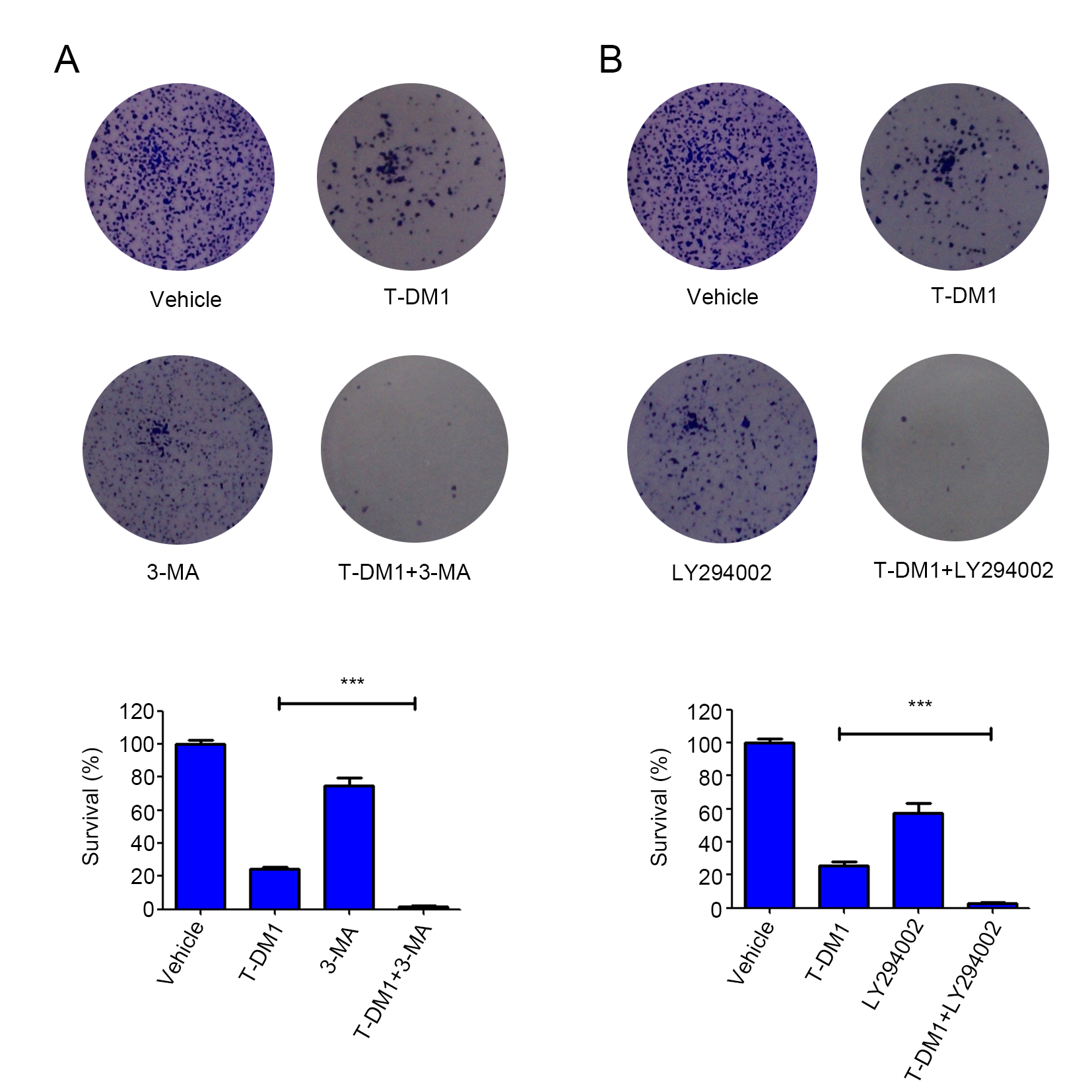

Supplement: Supplementary file 9 — Supplementary Figure S8 [file 41419_2020_3349_MOESM9_ESM.tif]

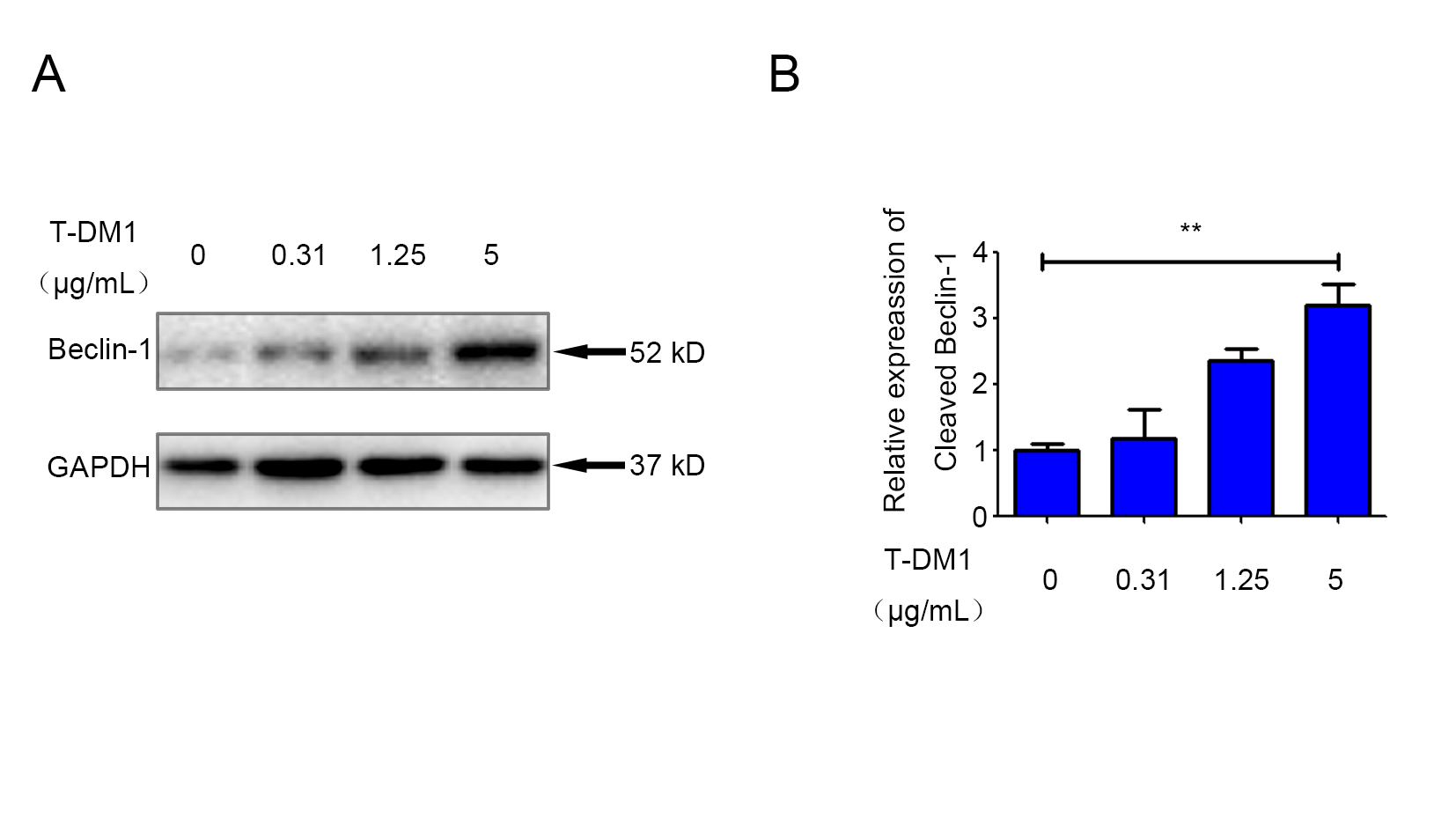

Supplement: Supplementary file 10 — Supplementary Figure S9 [file 41419_2020_3349_MOESM10_ESM.tif]

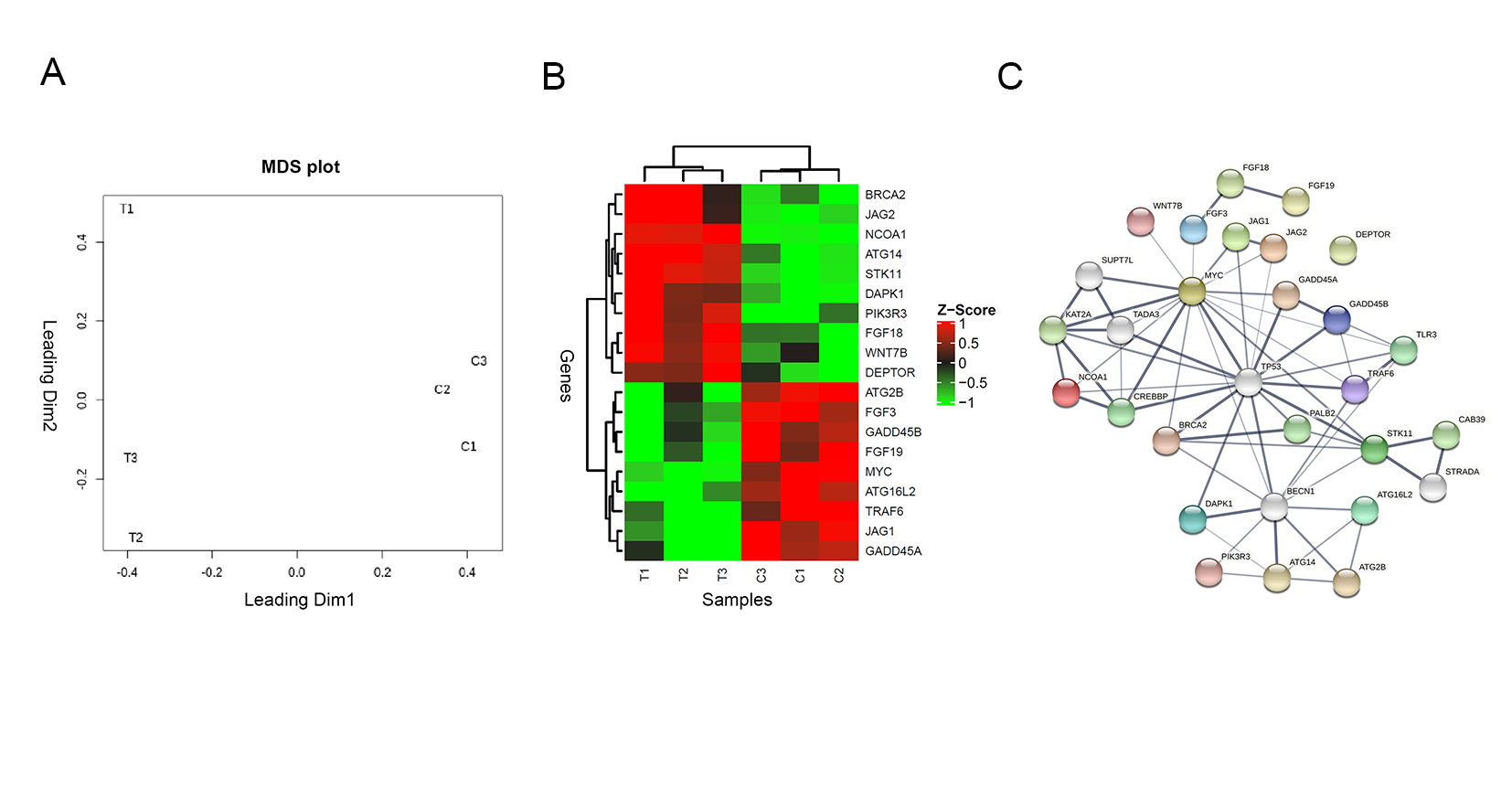

Supplement: Supplementary file 11 — Supplementary Figure S10 [file 41419_2020_3349_MOESM11_ESM.tif]
